# Identification of Interconnected Markers for T-Cell Acute Lymphoblastic Leukemia

**DOI:** 10.1155/2013/210253

**Published:** 2013-07-15

**Authors:** Emine Guven Maiorov, Ozlem Keskin, Ozden Hatirnaz Ng, Ugur Ozbek, Attila Gursoy

**Affiliations:** ^1^Center for Computational Biology and Bioinformatics and College of Engineering, Koç University, Rumelifeneri Yolu, Sariyer, 34450 Istanbul, Turkey; ^2^Department of Genetics, Institute for Experimental Medicine (DETAE), Istanbul University, 34393 Istanbul, Turkey

## Abstract

T-cell acute lymphoblastic leukemia (T-ALL) is a complex disease, resulting from proliferation of differentially arrested immature T cells. The molecular mechanisms and the genes involved in the generation of T-ALL remain largely undefined. In this study, we propose a set of genes to differentiate individuals with T-ALL from the nonleukemia/healthy ones and genes that are not differential themselves but interconnected with highly differentially expressed ones. We provide new suggestions for pathways involved in the cause of T-ALL and show that network-based classification techniques produce fewer genes with more meaningful and successful results than expression-based approaches. We have identified 19 significant subnetworks, containing 102 genes. The classification/prediction accuracies of subnetworks are considerably high, as high as 98%. Subnetworks contain 6 nondifferentially expressed genes, which could potentially participate in pathogenesis of T-ALL. Although these genes are not differential, they may serve as biomarkers if their loss/gain of function contributes to generation of T-ALL via SNPs. We conclude that transcription factors, zinc-ion-binding proteins, and tyrosine kinases are the important protein families to trigger T-ALL. These potential disease-causing genes in our subnetworks may serve as biomarkers, alternative to the traditional ones used for the diagnosis of T-ALL, and help understand the pathogenesis of the disease.

## 1. Introduction

T-lineage acute lymphoblastic leukemia (T-ALL) is known to result from malignant transformation of hematopoietic precursor cells at different maturation stages of T cells, the so-called thymocytes [1]. Proliferation of developmentally arrested T cells gives rise to T-ALL. Differentiation arrest may take place at almost all stages of thymocyte development [2]. T-ALLs are a heterogeneous set of diseases, in terms of cytogenetics, molecular aberrations, and clinical characteristics [1]. Its pathogenesis and subtypes are usually undefined. T-ALL constitutes 15% of pediatric and 25% of adult ALL cases [1–4]. 

In early stages of thymocyte differentiation, immature T cells undergo V(D)J recombination [1]. During this time, many other genes, especially the T-cell receptor (TCR) genes, are transcribed and are in “open chromatin” configuration, meaning that they are easily accessible to DNA binding proteins, like recombinases. An unusual recombinase action may lead to translocation of chromosomes [3, 5, 6]. Generally, the translocations involve abnormal juxtaposition of powerful enhancers or promoters of TCR genes with genes on other chromosomes, such as transcription factors or oncogenes [3, 7]. Translocations give rise to not only promoter exchange but also fusion genes, encoding chimeric proteins [1]. The other molecular-genetic abnormalities in T-ALL involve deletions, amplifications, and point mutations which activate oncogenes or inhibit tumor suppressors, which, in turn, cause differentiation arrest in thymocytes [1–3]. Deletions are the reason for loss of tumor suppressors. The correct diagnosis of acute leukemia requires wide-ranging diagnostic procedures together with cytochemistry, multiparameter flow cytometry, cytogenetics, fluorescence in situ hybridization, and molecular-genetic methods [8]. Although chromosomal rearrangements are common to T-ALL, there is still a large fraction of incidents (50%) where normal karyotype is seen [1]. 

The precise diagnosis of a tumor type is the most significant step in cancer treatments [9]. In order to apply the appropriate therapy, with maximum efficiency and minimum toxicity, the cancer should be diagnosed and classified correctly [10]. The challenge is to identify new diagnostic biomarkers to differentiate diseased and healthy individuals properly. An optimum biomarker would be easily analyzed by a single test and measurable in body fluids (such as blood or urine). However, cancer, in our case T-ALL, is a complex disease, and it is very difficult to find a single optimal biomarker at the molecular level [11].

The analysis of genome-wide expression profiles is frequently used to discover new biomarkers [12]. Since microarray or RNAseq high-throughput experiments give information about the expression of many genes in parallel [13], they are proposed to be a robust technology for the identification of signatures or expression patterns that vary significantly between diseased and healthy samples [11, 14, 15]. The current long-lasting diagnostic procedures for leukemia (e.g., cytomorphology, immunophenotyping, and metaphase cytogenetics) might be replaced by the comprehensive microarray or RNAseq protocols which takes two or fewer days and allows the simultaneous detection of the expression of almost all genome in one experimental approach [9]. With the extensive usage of microarrays, an increasing number of methods have been developed to identify biomarkers [14, 16–20].

Along with the advantages of microarrays, there are some limitations. For instance, some important genes of cancer are not differentially expressed at the level of transcription or the fate of cancer may not be controlled at the level of expression [11]. In addition, the transcriptional level does not always correlate with the translational level; in other words, the mRNA expression is not always equal to the protein expression [21]. The gene-expression levels may vary even in the genetically identical cells with the same histories of environmental exposure. These variations, known as “noise,” come from the random nature of biochemical reactions [22]. Moreover, there is also an “experimental noise” other than the “expression noise” (biological variations), in which slight unintended differences in experimental setup may lead to huge differences in hybridization of probes. This kind of technical noise is also considered as one of the restrictions for successful use of this technology [23–25]. Another limitation of microarrays might be the lack of pathway knowledge. One way to overcome this problem is to integrate gene-expression profiles with protein-protein interaction (PPI) networks [26–29]. A biological function or a phenotype is not controlled by just one gene [30]; rather pathways or cross-talks among proteins are responsible for the regulation of a function [31–33]. Thus, network information provides a functional insight when integrated with microarray data [11]. Therefore, identification of differential gene modules or subnetworks instead of individual differentially expressed genes may increase the reliability and robustness of biomarkers [11]. 

Traditional expression-based classification techniques identify only differentially expressed (DE) genes as signatures/markers, but a network-based approach returns subnetworks including both DE and non-DE genes [12]. The differential expression analysis of gene-expression profiles returns massive numbers of genes which makes it difficult to conclude that the differential expression of a particular gene has resulted from the disease/abnormality. Therefore, we cannot be so sure that the differential analysis identifies genes whose differential expression has only resulted from the disease: there may be another factor affecting the expression of that gene, such as experimental setup, treatment, or gender. As noted earlier, even cells with identical genome and environmental exposure history show variations in their gene expression (noise). Thus, just differential analysis alone is not reliable enough to conclude a gene as a biomarker of a disease.

However, network-based approaches do not depend on only expression data. They integrate microarray data with PPI data and return subnetworks rather than individual genes. The number of subnetworks is not as high as the number of individual genes identified by differential analysis. These subnetworks are differential as a whole, but individual genes in a subnetwork may not be differential. Although irresponsive genes cannot be regarded as biomarkers, they have very important role in interconnecting several DE genes and can help us to understand the pathogenesis of the disease. The non-DE genes are necessary to maintain the integrity of the subnetworks, meaning that they are required to interconnect highly DE genes. Furthermore, generation of a disease necessitates some mutations or polymorphisms that may lead to loss of function or gain of function of proteins without affecting the expression level of that protein. This does not mean that this particular protein is not significant for the disease just because it is not differentially expressed. The resulting subnetworks represent models of the underlying molecular mechanisms. Each module corresponds to a different functional pathway or complex. Since genes function in collaboration rather than alone, subnetworks are more rational than independent responsive genes, from a biological perspective [34].

In this study, we used PinnacleZ algorithm [12] to integrate microarray and PPI data. In addition to PinnacleZ algorithm, there are several other studies which provide different ways to integrate gene-expression data with other biological data, such as protein-protein interactions, protein-DNA interactions, molecular signatures, or hub proteins [35–42]. These integrated analyses not only improve the prediction accuracy but also shed light on the biological pathways involved in the pathogenesis of the diseases. However, there are not significant differences in their prediction accuracies among themselves [43]. Combining PPI and microarray data has led also to determining some important proteins that are highly connected in interaction networks: “party” and “date” hubs [44, 45]. Party hubs are the ones highly correlated with its interacting partner proteins (coexpressed) and date hubs are less-correlated genes with its interacting partners [44, 45]. The method of Taylor et al. [42] successfully used hub proteins to reveal the dynamic modularity in protein networks to predict breast cancer outcome.

Hierarchical clustering is one example of the expression-based classification techniques which groups together both genes and samples with similar expression patterns. It can also define subclasses of a disease, such as cancer (e.g., different stages of cancer or different types of cancer, like B-ALL versus T-ALL) [46]. Clustering algorithms try to organize genes or samples according to their similarity in expression. Genes with related pattern appear in the immediate vicinity of each other. In other words, it gathers coexpressed genes together. It also has a tendency to arrange genes with similar functions together since the functionally related genes are likely to be co-expressed [47]. Hierarchical clustering is a type of unsupervised clustering which does not use any prior knowledge regarding the sample classes.

## 2. Methods

### 2.1. Microarray Data (The MILE Study)

We used a comprehensive group of Affymetrix microarray datasets to determine which genes or modules discriminate T-ALL samples from healthy individual samples. This study included bone marrow samples of 173 T-ALL patients at diagnosis (untreated patients) and 74 nonleukemia/healthy specimens (e.g., healthy, hemolysis, and iron deficiency). The patient samples were heterogeneous; that is, there were samples from different stages of T-ALL. All microarray data were obtained from the Microarray Innovations in Leukemia (MILE) study, the National Center for Biotechnology Information's Gene Expression Omnibus database (http://www.ncbi.nlm.nih.gov/geo/) under series accession no. GSE13204. The MILE study is an international standardization program and was conducted by 11 laboratories across three continents [13]. The comprehensive MILE data has a high degree of intra- and interlaboratory correlation, meaning that they tried to minimize the disadvantages of microarrays, such as noise. In order to avoid the limitations of microarrays, we used only MILE data. There were two stages in this study containing microarray samples of 17 different classes of leukemia and myelodysplastic syndromes and an 18th class of non-leukemia. We used only the first stage in which the whole genome microarray platform (HG-U133 Plus 2.0; Affymetrix, Santa Clara, CA, USA) was used [15]. In the GEO-series matrix, the microarray data were already summarized and quantile normalized as described before [48].

In order to integrate microarray data with the human PPI network, it is necessary to convert Affymetrix probeset IDs to corresponding Entrez gene IDs. The annotation data was also provided under the same accession number. It is important to note that values for multiple probes corresponding to the same Entrez ID were averaged so that a particular gene ID is seen only once throughout the microarray data.

We also used a recently published dataset to test performance of our subnetworks. This dataset includes gene-expression profiles of childhood T-ALL bone marrow samples under series accession no. GSE46170 from GEO. This dataset includes 31 patients and 7 healthy samples.

### 2.2. Human Protein Interaction Network

The human PPI network is obtained from Human Protein Reference Database. There are 38788 PPIs whose interactions are experimentally verified and extracted from the literature [49].

### 2.3. Data Integration

In order to combine human PPI data with microarray data, we used a previously described algorithm (PinnacleZ, a plugin to cytoscape) [12]. This algorithm superimposes gene-expression values with corresponding network proteins, begins from every protein (seed) in the PPI network, and greedily appends interactions to identify the subnetwork starting from each seed whose mean expression for each sample best discriminates between the two sample types (In our case, the sample types are T-ALL and nonleukemia/healthy conditions). At first, the number of resulting subnetworks is equal to the number of proteins in the PPI network. Then, nonsignificant modules are filtered out by three types of permutation testing [30]. At last, subnetworks with high discriminative potential are obtained.

To equate the number of healthy individuals (74) to the number of patients with T-ALL (173), we divided T-ALL patient samples into two groups randomly. Then, we merged each half of patient data with the healthy data, separately. For each merged data (87 + 74 and 86 + 74), we ran the algorithm 4 times. Then, we resampled our patient samples 5 times (we again divided T-ALL samples into 2 groups randomly; thus these 2 groups are different from the 2 groups created before. So, we generated 10 different combinations of patients) and repeated the merging and algorithm-running steps. We obtained results of 40 runs in total.

### 2.4. Classification Accuracy

The classification accuracy of a given subnetwork or the overall rate of correct predictions of the patient and healthy datasets was estimated by 10-fold cross-validation with two different classifiers (J48 and RBF network classifiers implemented in WEKA [50]). According to 10-fold cross-validation, the complete dataset was divided into 10 uniformly sized subgroups; the classifier was trained for nine subgroups, and predictions were made for the remaining subgroup. High classification accuracy, like 90%, means that a given subnetwork differentiates/predicts the patient and healthy samples correctly, 90% of the time.

As pointed out, we randomly divided diseased samples into two groups: for each half we found subnetworks and determined their classification accuracy. We used the other half as a validation set to cross-test the prediction accuracy of a given subnetwork.

We also used an independent microarray dataset (childhood T-ALL samples, GSE46170) to test the classification accuracies of our subnetworks. Again the prediction accuracies are obtained by J48 and RBF network classifiers in WEKA, by 10-fold cross-validation.

### 2.5. Functional Enrichments

Functional enrichment analysis was achieved by using Gene Ontology Tree Machine (GOTM) which searches for functional enrichments from Gene Ontology (GO) and Kyoto Encyclopedia of Genes and Genomes (KEGG) categories for genes in the given subnetworks.

### 2.6. Hierarchical Clustering

In order to identify groups of genes that have similar expression patterns and to show the difference between expression-based and network-based classification approaches, hierarchical clustering method was applied to 173 T-ALL and 74 healthy samples together. Initially, there were 20148 probes. Then, these probes are filtered by *t*-test. We determined the first 100 most differential genes, and we repeated the same analysis with the first 200 most differential genes. The rationale behind this is to analyze how the results would change between these two sets. Hierarchical clustering algorithm which is implemented in Expander [51] was performed on both the 200 and 100 most differentially expressed genes. Both samples and genes are clustered with complete linkage and Pearson correlation.

## 3. Results and Discussion

We applied both expression-based and network-based approaches to find important genes for the generation of T-ALL and to show that network-based approaches are more successful in returning more meaningful results than expression-based approaches.

As a network-based approach, we used PinnacleZ algorithm [12] to distinguish T-ALL patients and healthy samples by integrating microarray data with the human PPI network. This approach enabled us to identify subnetworks/modules as markers which differentiate the patients from healthy individuals. The size of subnetworks or the number of genes in a subnetwork varies, ranging from 1 to 10 genes. As noted earlier, the individual genes may not be responsive, meaning that expression of a particular gene is different in patients and healthy samples, but an entire subnetwork is differential.

**Figure 1:**
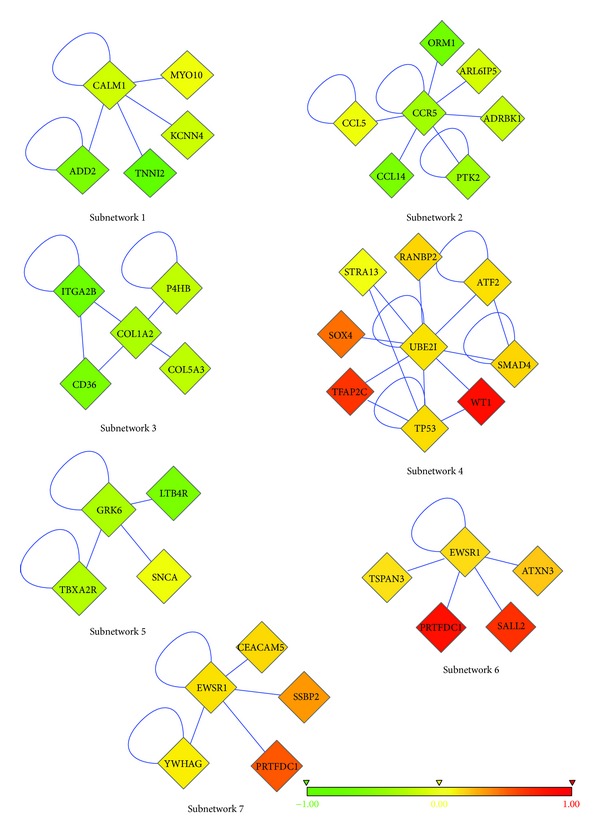
First seven of the most frequent subnetworks. Nodes represent proteins, and edges represent interactions. The color of each node ranges in accordance with the change in expression of the corresponding gene for T-ALL versus healthy samples. The shape of each node shows whether its gene is significantly differentially expressed (diamond; *P* < 0.05 from a two-tailed *t*-test) or not (circle).

**Figure 2:**
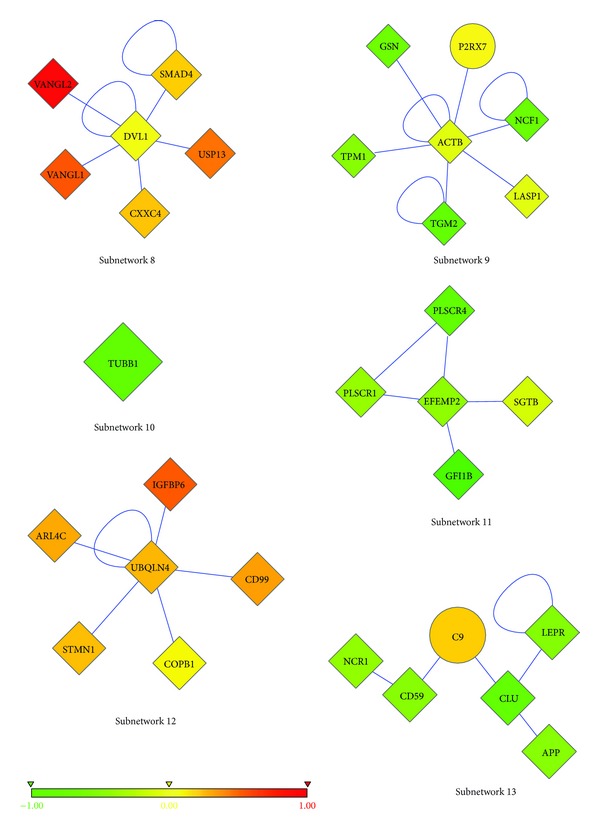
Second six of the most frequent subnetworks. Nodes represent proteins, and edges represent interactions. The color of each node ranges in accordance with the change in expression of the corresponding gene for T-ALL versus healthy samples. The shape of each node shows whether its gene is significantly differentially expressed (diamond; *P* < 0.05 from a two-tailed *t*-test) or not (circle).

**Figure 3:**
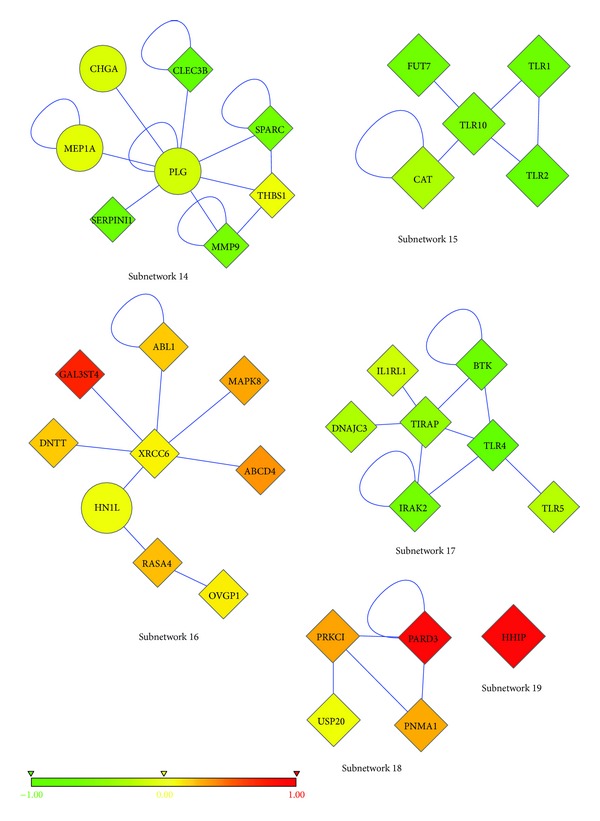
The last six of the most frequent subnetworks. Nodes represent proteins, and edges represent interactions. The color of each node ranges in accordance with the change in expression of the corresponding gene for T-ALL versus healthy samples. The shape of each node shows whether its gene is significantly differentially expressed (diamond; *P* < 0.05 from a two-tailed *t*-test) or not (circle).

As explained in the Methods section, we prepared different combinations (10 different combinations) of patient-healthy merged data and ran the algorithm 4 times with each combination. Then, we focused on the most frequent subnetworks. We recovered the most repeated 19 subnetworks, out of 183 subnetworks (see Figures 1, 2, and 3. Please refer to Supplementary Material available online at http://dx.doi.org/10.1155/2013/210253 (Table S1–Table S19) to see the names and functions of these genes.). These subnetworks cover 102 genes in total, and the majority of them are differentially expressed (DE). There are only 6 genes whose expression is not differential among T-ALL and healthy samples (see Table 1). The list of genes in the subnetworks, their functions, and the pathways they are involved in can be found in Table 2 and also in the Supplementary Material.

Development of cancer requires the accumulation of several mutations in several genes in different pathways [52]. Since the cell regulation is controlled by many pathways, a mutation in one pathway may be compensated by other pathways. But if there are many mutations in numerous pathways, the harmful impacts of these mutations cannot be compensated. For instance, if a tumor suppressor gene is inactivated, it is not enough to generate cancer; additional mutations are needed before cancer appears [53]. We found that the subnetworks correspond to different pathways, in general (Table 2). This result supports the notion that multiple cell regulatory pathways are involved in production of leukemogenesis. Targeting defective molecular pathways is more effective in getting rid of cancer cells and less destructive for rapidly dividing normal cells, which is referred to as “targeted therapy” [54, 55]. Since targeted therapy aims at destroying only tumor cells whose particular pathways are broken or malfunctioning, it might have fewer side effects than chemotherapy and radiotherapy which are cytotoxic to all fast-proliferating cells, including the healthy ones. Targeting our subnetworks or their corresponding pathways may result in development of efficient novel drugs for T-ALL treatment.

Among the genes in the subnetworks, some are known to be associated with T-ALL from previous studies: ABL1 [1, 56, 57], CCL5 [58], CD99 [59], TP53 [60], and WT1 [61, 62].  Table 3 exhibits these T-ALL related genes and their behavior. Many other genes seen in subnetworks were not previously found to be linked to T-ALL (some are related to cancer, but not specifically to T-ALL, such as VANGL1 [63], CEACAM5 [64], SSBP2 [65], LTBR4 [66], TFAP2C [67], USP13 [68], CD36 [69], UBE2I [70], EWSR1 [71], SOX4 [72], and LASP1 [73]) (See Table 4, for corresponding subnetworks and the behavior of these genes). So, after experimental validation, these genes may serve as novel markers for T-ALL.

There are 6 non-DE genes in our subnetworks (Table 1). Although these irresponsive genes cannot be considered as markers of T-ALL, they have very important roles in interconnecting numerous DE genes, and their presence could be essential for malignant transformation of precursor T-cells. One of the 6 non-DE genes is P2RX7 (Figure 2, subnetwork 9), which is a purinergic receptor P2X, expressed in hematopoietic cells, and mediates both apoptosis and proliferation, depending on the level of activation [74–76]. Prolonged activation of this receptor by extracellular ATP is a significant mechanism to initiate apoptosis in T and B lymphocytes [76, 77]. Its loss of function by an SNP (1513A→C) has an antiapoptotic effect and is previously shown to be related to chronic lymphoblastic leukemia (CLL). Although this SNP abolishes the function of P2RX7, it does not have an impact on the expression level of the receptor [76, 78]. Thus, loss of function does not imply reduced expression. In T-ALL, it is possible seeing a similar mutation which leads to loss of function of this receptor, without affecting its expression level. Therefore, despite its nondifferential expression, it may contribute to the pathogenesis of the disease. Only differential expression analysis would not highlight this gene as important, but network-based approach detected it as a significant one. Moreover, loss of function of P2RX7 decreases the efficiency of adjuvant chemotherapy in breast cancer patients [79]. So, P2RX7 should be present to benefit from chemotherapy. Apart from its role in apoptosis, it also promotes proliferation upon weak stimulation [80]. Furthermore, P2RX7 expression (not necessarily upregulation) results in increased proliferation and reduced apoptosis [81]. When oxidized ATP, P2RX7 inhibitor, was injected into the tumor, the tumor shrank [81]. Upregulation of this gene was seen in acute lymphoblastic leukemia (ALL), acute myelogenous leukemia (AML), chronic myelogenous leukemia (CML), and myelodysplastic syndrome [82]. In addition, its high expression diminished the remission rate of AML after a dose of standard therapy [82]. Although the upregulation of P2RX7 is not observed in our T-ALL samples, these data suggest that it is worthwhile to further investigate the potential role of P2RX7 in the generation of T-ALL.

PLG and MEP1A are other examples of our non-DE genes. PLG (Figure 3, subnetwork 14) encodes plasminogen which is essential for cancer cell invasion and metastasis. Plasminogen activators convert plasminogen to active plasmin which in turn activates MEP1A [83]. MEP1A (Figure 3, subnetwork 14), meprin A, is a metalloprotease that cleaves proteins and degrades extracellular matrix, facilitating the tissue invasion and metastasis [84]. They participate in the migration of leukocytes to the sites of infection and migration of cancer cells in metastasis [85]. Meprin A is upregulated in several cancer cells [86, 87]. T-cell lymphoid tumor growth is decreased by plasmin inhibitors by suppressing metalloproteinases [88]. Even though these two genes are not DE in our T-ALL samples, there is clear evidence that these two groups of enzymes are very important candidates of disease-causing genes.

Another non-DE gene is CHGA, chromogranin A, which is an acidic glycoprotein commonly expressed by neuroendocrine cells [89]. It is widely used as a diagnostic and prognostic biomarker for neuroendocrine tumors [90]. Our fifth non-DE gene is C9, complement component 9. Tumor cells possess some protective mechanisms against complement-mediated tumor cell lysis [91]. Human leukemic cells remove membrane attack complexes from their surfaces by phosphorylating C9 [92]. Therefore extracellular phosphorylation of C9 provides a defense mechanism against complement system. In addition, complement system is defective in CLL patients [93]. T-ALL could have a similar protective method as CLL does. Further studies are necessary to elucidate the roles of these non-DE genes in the pathogenesis of T-ALL. These genes may lead to new clinical therapies for T-ALL.

Subnetworks are rich in transcription factors: there are 14 transcription factors involved in subnetworks (Table 5). This result is in accordance with the important assumption that abnormal or ectopic activation of specific transcription factor genes, with/without chromosomal rearrangements, is the main event in transformation of immature T cells [5].

There were 6 tyrosine kinases (Table 6), in our subnetworks. Tyrosine kinases have a critical role in TCR signaling, regulation of T-cell immune response, and T-cell survival and proliferation. Expression of tyrosine kinases, like ABL1, affect pre-TCR and TCR signaling and give a proliferative and survival advantage [1]. ABL1 is an oncogene and is often seen as ABL1-NUP214 fusion gene in T-ALL [1, 7, 61]. As can be seen in subnetwork 16 (Figure 3), ABL1 is upregulated in T-ALL patients compared to healthy individuals. Although YWHAG was not found to be related to T-ALL before, it interacts with ABL1. The overexpression of ABL1 might induce upregulation of the YWHAG gene (Figure 1, subnetwork 7). BTK is significantly downregulated in T lymphocytes, which is consistent with our results. PTK2, also known as FAK, Focal Adhesion Kinase, (Figure 1, subnetwork 2) has a role in growth, differentiation, tumor metastasis, and wound healing [94–97]. Its overexpression is associated with several types of cancer [98, 99]. But in a recent study, it has been shown that PTK2 protein is predominantly absent in both normal T cells and T-lymphoblastic leukemia/lymphoma. Although it is negative in T-cell leukemia/lymphoma, it is mostly positive in B-cell lymphomas [96]. Consistent with the literature, PTK2 gene is also downregulated in T-ALL patients used in this study. Cell adhesion molecules (CAMs) are necessary for interaction of hematopoietic cells with extracellular matrix with stromal and other cells [53]. Defects in adhesion were reported in other types of leukemia before, such as in chronic myeloid leukemia (CML) [53, 100, 101]. The failure of hematopoietic stem cells (HSCs) to express the correct or fundamental adhesion molecules may contribute to transformation of a normal HSC to leukemic cell and to get arrested at a particular step of their differentiation. The adhesion deficiency may also help leukemic cells to escape from the recognition by immune system [53].

Interestingly, the subnetworks are also abundant in zinc-ion (Zn^2+^) binding proteins (Table 7) which are generally enzymes, including those involved in DNA repair. Zinc-finger motifs play key role in interaction of proteins with nucleic acids (DNA/RNA) [102]. They are essential for site-specific DNA recognition and transcriptional activation [103]. Zn^2+^ has both structural and regulatory roles in zinc-binding proteins, meaning that Zn^2+^ maintains the three-dimensional structure of the proteins, and it is required for the proper function of the proteins. For example, p53 needs Zn^2+^ to fold properly. Both excess and inadequate amounts of Zn^2+^ cause misfolding of p53 [103]. One of the molecular mechanisms in carcinogenesis is the deformation of zinc-finger domains in DNA repair proteins [102]. Zn^2+^ is also important for thymic immune responses [104]. Low levels of zinc are frequently reported in ALL cases. Normal lymphocytes contain more zinc than leukemic cells [105]. Treatment of ALL patients with zinc, in addition to chemotherapy, was hypothesized to increase the overall ability to recover from T-ALL permanently and to endure toxic effects of chemotherapy [106]. Some of the Zn^2+^ ion binding proteins are upregulated, but some are downregulated (see Table 7). Although zinc-ion binding proteins do not behave similarly at the level of expression (i.e., they are not all up-regulated or down-regulated: some of them are upregulated, while some are downregulated), it is evident that more attention should be paid to them.

Two subnetworks are actually individual genes rather than interconnected genes (subnetwork 10, Figure 2 and subnetwork 19, Figure 3). These genes are TUBB1 and HHIP. HHIP stands for Hedgehog Interacting Protein, which is a negative regulator of Hedgehog signaling pathway. Overactivity of Hedgehog signaling pathway is related to many cancer types [107]. HHIP is found to be associated with lung cancer [108] and brain tumor [109]. Although it is down-regulated in several tumor types, it is up-regulated in our T-ALL patients (subnetwork 19, Figure 3) [110, 111]. The other individual gene that is found as a marker is TUBB1, Tubulin beta-1 (subnetwork 10, Figure 2), which has a role in assembly of microtubules only in hematopoietic cells. Altered expressions of beta-tubulin isotypes were observed in specific tumor types [112]. Tubulin mutations are involved in resistance to drugs that target microtubules in cancer patients [113]. 

CALM1 gene, a member of subnetwork 1 (Figure 1), has been shown to be involved in a translocation with AF10 gene, and this fusion gene was detected in almost 10% of immature T-ALL patients. CALM-AF10 fusion gene upregulates HOXA gene cluster and has shown to be related to bad prognosis [114]. Dik et al. studied gene-expression profiles of CALM-AF10 positive and negative T-ALL patients and revealed that the TUBB gene was 7-fold overexpressed in CALM-AF10 positive patients [115]. In this study, TUBB1 gene was detected in subnetwork 10 as an individual gene (Figure 2). TUBB polymorphisms were described in ALL patients, and they were suspected to be involved in drug resistance [116]. ADD2 gene is another gene in subnetwork 1. ADD genes are a family of cytoskeleton proteins encoded by three genes (ADD1, ADD2, and ADD3). ADD2 gene knockout mice are used as models for leukemia, and ADD3 gene was shown to have a translocation with NUP98 in T-ALL patients [117]. These two findings show that ADD gene family takes place in the hematopoiesis and also in hematologic malignancies. 

In subnetwork 8 (Figure 2), two genes that take part in early developmental stages, VANGL1 and VANGL2, are found directly related to DVL gene. DVL gene negatively regulates WNT signalling pathway which plays an important role in the hematopoiesis, particularly in T-cell development [118]. These findings stress once again the importance of these networks in T-ALL pathogenesis not only on gene-expression level but also on protein level. 

### 3.1. Classification Accuracies of Subnetworks

After finding subnetworks, we tested their classification accuracies with two different classifiers, namely, J48 and RBF-network in WEKA [50]. The prediction accuracy of each subnetwork was tested individually with 10-fold cross-validation. As discussed, the patient samples were randomly divided into 2 groups, and, for each subgroup, differential subnetworks were found. Classification accuracies of subnetworks were found by testing their original sub-group (the group for which the subnetworks were found) and by cross-testing the remaining sub-group (the other half of the patients). The cross-testing was applied to validate the prediction accuracies of modules also in different sets of patient microarray data. All of the subnetworks achieved very high accuracies in prediction, higher than 90% (Table 8). There are some subnetworks that achieved 99% prediction accuracy (Figure 1, subnetwork 4 and Figure 2, subnetwork 12). These results also prove the success of the network-based classification approaches. Cross-comparisons between two independent halves of patient dataset revealed that subnetworks are good at distinguishing T-ALL patients from healthy individuals, regardless of the dataset in which they are found. In other words, they can classify patients in both independent datasets with similar accuracies.

To check whether our subnetworks are also applicable to other publically available microarray data, we used gene-expression profiles of childhood T-ALL samples (GSE46170). As Table 9 shows, the classification accuracies of subnetworks on this independent dataset are also relatively high, about 83% on average (ranging 71–94%) over subnetworks. Compared to original dataset (MILE) on which the subnetworks are found, the independent dataset showed lower performance on classification. This decrease in classification accuracies may stem from the fact that the independent dataset contains only childhood T-ALL samples as opposed to MILE study which has heterogeneous patients, meaning that there are patients from different stages of the disease and they are not specifically childhood T-ALL samples. Another reason may be the imbalanced number of patients (31 patients) and healthy samples (7 healthy individuals) in this independent dataset. The imbalanced numbers of healthy and patient samples may also decrease the prediction accuracy.

Moreover, to demonstrate the accomplishment of our subnetworks, we compared them with T-ALL related genes in KEGG pathways [119], considered as a module.  Table 10 displays these 21 genes, and Table 11 presents their classification accuracies. The performance of these genes is also high, but this outcome is not surprising because these 21 genes are already known to be related to T-ALL. However, our subnetworks largely consist of novel markers of T-ALL, and they do the same or better jobs than these 21 genes in KEGG pathway. Moreover, there is also a decrease in accuracies of these genes when tested on independent dataset compared to MILE dataset. So, it is normal that our subnetworks achieve higher accuracies in MILE dataset but lower in the independent dataset (GSE46170). As indicated above, the reason may be the imbalanced numbers of healthy and patient samples.

Although integrating microarray data with network information is a promising way to identify functional biomarkers, the drawback of pathway-based classifiers is that most of the human genes have not been assigned to a definitive pathway yet [12]. As pathways become more complete, the classification performances of pathway-based approaches will increase [18].

### 3.2. Hierarchical Clustering Results

**Figure 4:**
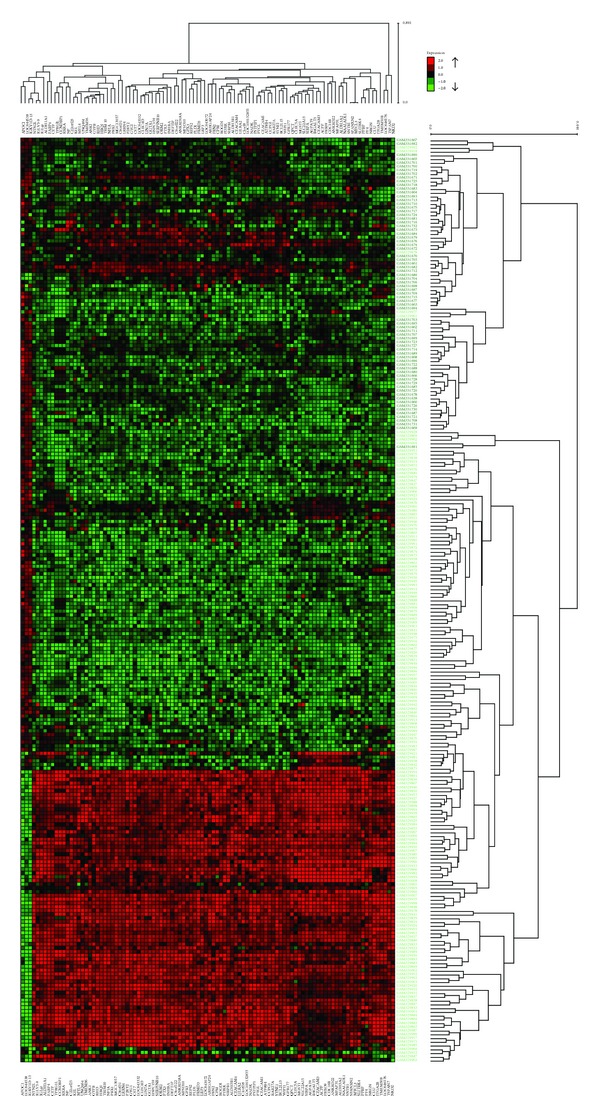
The heat map shows the hierarchical clustering result of the 100 most differentially expressed genes in T-ALL with respect to healthy individuals. Red and green spots represent upregulated and downregulated genes, respectively. Black spots denote equal expression. The columns labeled with light green belong to healthy individuals, and the columns labeled with black are individuals with T-ALL.

**Figure 5:**
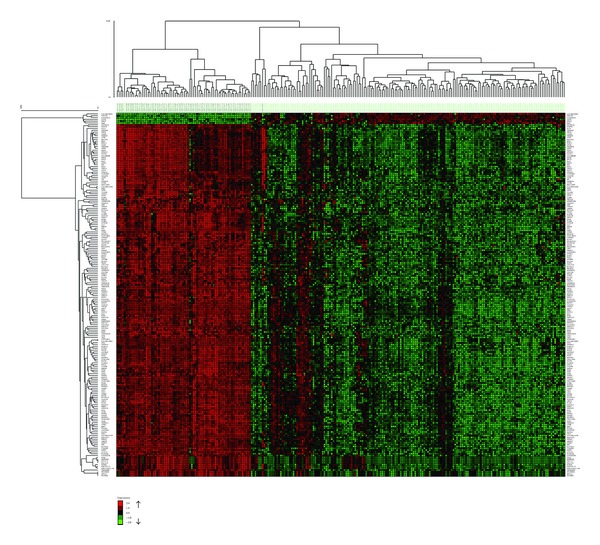
The heat map shows the hierarchical clustering result of the 200 most differentially expressed genes in T-ALL with respect to healthy individuals. Red and green spots represent up-regulated and down-regulated genes, respectively. Black spots denote equal expression. The columns labeled with light green belong to healthy individuals, and the columns labeled with black are individuals with T-ALL.

After filtering 20148 probes in microarray data with *t*-test and obtaining the 100 and 200 most DE genes, hierarchical clustering was applied with complete linkage and Pearson correlation. The resulting clusters are shown in Figures 4 and 5. Since there are too many samples and genes, the gene names are not visible on these figures. Please refer to Supplementary Material (Table S20 and Table S21) to see the names and functions of these genes.

In Figure 4, cluster results of the 100 most DE genes, 5 T-ALL samples, and 1 healthy sample are misclassified, meaning that 5 T-ALL samples were grouped in healthy samples and 1 healthy sample was grouped in patient samples. Moreover, there are 1 T-ALL and 1 healthy sample, which are misclassified in clusters of the 200 most DE (Figure 5). This result is expected since 200 genes provide more information. However, the number of genes is much larger than the one we obtained in subnetworks. 

 A far more striking result is that 102 genes in our subnetworks and the 100 most DE genes have only 2 genes in common (Table 12). Furthermore, subnetworks and the 200 most DE genes have only 8 genes in common (Table 13). This result shows that 94 of our subnetwork genes are less differential than the 200 most DE genes. There are also 6 non-DE genes in our subnetworks (in the remaining 94 genes). Therefore, it would not be wrong to expect much higher classification accuracies from the 100 and 200 most DE genes than that of 102 subnetwork genes. But the classification accuracies of the 100 and 200 most DE genes are not very different from those of subnetworks. Actually, 2 subnetworks (subnetworks 12 and 13, Figure 2) performed even higher classification accuracies than the 100 and 200 most DE genes.  Table 14 displays the classification accuracies of the 100 and 200 most DE genes. Thus, we can safely conclude that our subnetworks (even they are less differential than the 100 and 200 most DE genes and contain non-DE genes) can do the same or better job in distinguishing diseased samples from healthy samples. Doing the same job with 10 genes, instead of 100 or 200 genes, might be regarded as an accomplishment. 

 It is interesting that very well-known cancer genes such as TP53 and MAPK8 were not included in the 100/200 most DE genes, but we were able to detect them by network-based approach.

In conclusion, each subnetwork with high prediction accuracy provides a new suggestion for pathways and molecular mechanisms involved in the pathogenesis of T-ALL. All subnetworks serve as a biomarker which can be helpful in diagnosis and in identifying potential drug targets for T-ALL, in the near feature. The accomplishment of network-based classification and subnetwork/pathway detection is in line with the idea that cancer is not a result of solely one pathway, but instead it is a “disease of pathways” [12, 52, 120]. Unlike conventional differential expression analysis, network-based approach allowed us to identify potential disease-causing non-DE genes. According to our results, we conclude that transcription factors, tyrosine kinases, and zinc-ion binding proteins are the most important protein groups involved in generation of T-ALL. 

The goal of this study is to highlight potential disease-causing genes for further experimental validation. It is beyond the scope of this study to verify all genes that appear in subnetworks; experimental proof is vital. We recommend investigators with an interest in a subnetwork/pathway to validate them with experimental techniques, like RT-PCR and Western blot. The important point of this work is that a combination of bioinformatic methods and high-throughput gene expression profiles and interactomics provide a promising way of identifying T-ALL specific modules and reveal pathways involved in T-ALL.

## Supplementary Material

The Supplementary Material contains 21 tables. The genes in the subnetworks and their functions are listed in Tables S1 through S19 (for each individual subnetwork, there is one table). Tables S20 and S21 display titles and functions of the 100 and 200 most differentially expressed genes.

## Figures and Tables

**Table 1:** GO enrichments of nondifferentially expressed genes.

Gene symbol	Subnetwork	Gene title	GO: function	GO: process
P2RX7	Sub-9	Purinergic receptor P2X, ligand-gated ion channel, 7	ATP binding, ATP-gated cation channel activity, ion channel, and receptor activity	Ion transport, signal transduction

C9	Sub-13	Complement component 9	No enrichments	Caspase activation, complement activation, and induction of apoptosis

CHGA	Sub-14	Chromogranin A (parathyroid secretory protein 1)	Calcium ion binding, protein binding	Regulation of blood pressure

PLG	Sub-14	Plasminogen	Apolipoprotein binding, calcium ion binding, peptidase activity, and plasmin activity	Blood coagulation, induction of apoptosis, negative regulation of angiogenesis, cell proliferation, fibrinolysis, proteolysis, and tissue remodeling

MEP1A	Sub-14	Meprin A, alpha (PABA peptide hydrolase)	Astacin activity, metal-ion binding, metallopeptidase activity, and zinc-ion binding	Digestion, proteolysis

HN1L	Sub-16	Hematological and neurological expressed 1-like	No enrichments	No enrichments

**Table 2:** KEGG and GO enrichments of the most frequent 19 subnetworks.

Subnetworks	KEGG	GO: function
Sub-1	No enrichments	Actin binding, calmodulin binding
Sub-2	Chemokine-signaling pathway, Chemokine-cytokine receptor interaction, endocytosis	Signal-transducer activity, chemokine activity, and cytokine activity
Sub-3	ECM-receptor interaction, focal adhesion, and hematopoietic cell lineage	Growth factor binding, extracellular matrix structural constituent
Sub-4	Pathways in cancer, colorectal cancer, pancreatic cancer, chronic myeloid leukemia, cell cycle, Wnt signaling pathway, and MAPK signaling pathway	Promoter binding, transcription activator activity, and transcription factor activity
Sub-5	Neuroactive ligand-receptor interaction	Signal-transducer activity, G-protein coupled receptor activity
Sub-6	No enrichments	No enrichments
Sub-7	No enrichments	No enrichments
Sub-8	Pathways in cancer, colorectal cancer, and Wnt signaling pathway	No enrichments
Sub-9	Leukocyte transendothelial migration, regulation of actin cytoskeleton, dilated cardiomyopathy, Fc gamma R-mediated phagocytosis, and hypertrophic cardiomyopathy (HCM)	GTP binding, actin binding
Sub-10	Phagosome, gap junction	GTP binding, GTPase activity, nucleotide binding, and structural molecule activity
Sub-11	No enrichments	Metal-ion binding, calcium ion binding, SH3 domain binding, and phospholipid transporter activity
Sub-12	No enrichments	No enrichments
Sub-13	Complement and coagulation cascades	No enrichments
Sub-14	Bladder cancer	Calcium ion binding, metalloendopeptidase activity
Sub-15	Toll-like receptor signaling pathway	Transmembrane receptor activity
Sub-16	Pathways in cancer, neurotrophin signaling pathway, ErbB signaling pathway, and nonhomologous endjoining	ATP binding, protein C-terminus binding
Sub-17	Toll-like receptor signaling pathway, pathogenic *Escherichia coli* infection	Transmembrane receptor activity
Sub-18	Tight junction, endocytosis	No enrichments
Sub-19	No enrichments	No enrichments

**Table 3:** T-ALL related genes found in subnetworks and their behavior in our samples.

T-ALL related genes	Subnetwork	Behavior
ABL1	16	↑
CCL5	2	↓
CD99	12	↑
TP53	4	↑
WT1	4	↑

**Table 4:** Cancer related genes recovered in subnetworks and their behavior in our samples.

Cancer related genes	Subnetwork	Behavior
VANGL1	8	↑
CEACAM5	7	↑
SSBP2	7	↑
LTB4R	5	↓
TFAP2C	4	↑
USP13	8	↑
CD36	3	↓
UBE2I	4	↑
EWSR1	6	↑
SOX4	4	↑
LASP1	9	↓

**Table 5:** Transcription factors recovered in subnetworks.

Gene symbol	Gene title	Subnetwork	Behavior
TNNI2	Troponin I type 2 (skeletal, fast)	Sub-1	Down
SMAD4	SMAD family member 4	Sub-4	Up
TP53	Tumor protein p53	Sub-4	Up
ATF2	Activating transcription factor 2	Sub-4	Up
WT1	Wilms, tumor 1	Sub-4	Up
SOX4	SRY- (sex determining region Y-) box 4	Sub-4	Up
TFAP2C	Transcription factor AP-2 gamma (activating enhancer binding protein 2 gamma)	Sub-4	Up
ATXN3	Ataxin 3	Sub-6	Up
SALL2	Sal-like 2 (*Drosophila*)	Sub-6	Up
EWSR1	Ewing sarcoma breakpoint region 1	Sub-6, sub-7	Up
SSBP2	Single-stranded DNA binding protein 2	Sub-7	Up
GFI1B	Growth factor independent 1B transcription repressor	Sub-11	Down
ABL1	c-abl oncogene 1, receptor tyrosine kinase	Sub-16	Up
XRCC6	X-ray repair complementing defective repair in Chinese hamster cells 6 (Ku autoantigen, 70 kDa)	Sub-16	Up

**Table 6:** Genes involved in tyrosine-kinase signaling pathway recovered in subnetworks.

Gene symbol	Gene title	Subnetwork	Behavior
BTK	Bruton agammaglobulinemia tyrosine kinase	Sub-17	Down
ABL1	c-abl oncogene 1, receptor tyrosine kinase	Sub-16	Up
YWHAG	Tyrosine 3-monooxygenase/tryptophan 5-monooxygenase activation protein, gamma polypeptide	Sub-7	Up
PTK2	PTK2 protein tyrosine kinase 2	Sub-2	Down
COL1A2	Collagen, type I, and alpha 2	Sub-3	Down
SPARC	Secreted protein, acidic, and cysteine rich (osteonectin)	Sub-14	Down

**Table 7:** Zinc-ion binding proteins recovered in subnetworks.

Gene symbol	Gene title	Subnetwork	Behavior
TP53	Tumor protein p53	Sub-4	Up
ATF2	Activating transcription factor 2	Sub-4	Up
WT1	Wilms, tumor 1	Sub-4	Up
SALL2	Sal-like 2 (*Drosophila*)	Sub-6	Up
EWSR1	Ewing sarcoma breakpoint region 1	Sub-6, sub-7	Up
USP13	Ubiquitin specific peptidase 13 (isopeptidase T-3)	Sub-8	Up
CXXC4	CXXC finger 4	Sub-8	Up
LASP1	LIM and SH3 protein 1	Sub-9	Down
GFI1B	Growth factor independent 1B transcription repressor	Sub-11	Down
APP	Amyloid beta (A4) precursor protein (peptidase nexin-II, Alzheimer's disease)	Sub-13	Down
MEP1A	Meprin A, alpha (PABA peptide hydrolase)	Sub-14	Non-DE
MMP9	Matrix metallopeptidase 9 (gelatinase B, 92 kDa gelatinase, and 92 kDa type IV collagenase)	Sub-14	Down
RASA4	RAS p21 protein activator 4	Sub-16	Up
BTK	Bruton agammaglobulinemia tyrosine kinase	Sub-17	Down
USP20	Ubiquitin specific peptidase 20	Sub-18	Up
PRKCI	Protein kinase C, iota	Sub-18	Up

**Table 8:** Classification accuracies of subnetworks found by two different classifiers in WEKA.

Subnetworks	J48	RBF network	J48 (validation set)	RBF network (validation set)
Sub-1	93	97	95	96
Sub-2	91	96	91	98
Sub-3	93	97	96	98
Sub-4	98.75	99	93	96
Sub-5	94	97	92	98
Sub-6	96	97	94	94
Sub-7	96	97.5	96	96
Sub-8	94	97	96	93.75
Sub-9	93	98	93	94
Sub-10	97	97	96	96.25
Sub-11	91	98	93	97
Sub-12	95	99	96	98
Sub-13	98	98	95	99
Sub-14	94	97.5	91	93
Sub-15	93	97	92	93
Sub-16	95	96	92.5	97
Sub-17	89	97	90	94
Sub-18	93	96	94	97
Sub-19	96.27	96.89	96.25	97

**Table 9:** Classification accuracies of subnetworks on an independent microarray data by two different classifiers in WEKA.

Subnetworks	J48	RBF network
Sub-1	73.68	76.31
Sub-2	94.73	92.1
Sub-3	81.57	76.31
Sub-4	68.42	94.73
Sub-5	89.47	97.36
Sub-6	78.94	78.94
Sub-7	78.94	81.57
Sub-8	89.47	84.21
Sub-9	89.47	81.57
Sub-10	81.57	73.68
Sub-11	81.57	86.84
Sub-12	68.42	89.47
Sub-13	71.05	71.05
Sub-14	81.57	89.47
Sub-15	92.1	86.84
Sub-16	86.84	86.84
Sub-17	84.21	89.47
Sub-18	92.1	81.57
Sub-19	81.57	76.31

**Table 10:** T-ALL related genes in KEGG pathways [119].

Gene symbol	Gene title
SIX4	SIX homeobox 4
LMO2	LIM domain only 2 (rhombotin-like 1)
HPGD	15-Hydroxyprostaglandin dehydrogenase (NAD)
GRIA3	Glutamate receptor, ionotropic, and AMPA 3
EYA1	Eyes absent homolog 1 (*Drosophila) *
FUT8	Fucosyltransferase 8 (alpha (1,6) fucosyltransferase)
MLL	Myeloid/lymphoid or mixed-lineage leukemia (trithorax homolog, *Drosophila*)
MEIS1	Meis homeobox 1
HOXA9	Homeobox A9
TLX1	T-cell leukemia homeobox 1
CCR7	Chemokine (C-C motif) receptor 7
LDB1	LIM domain binding 1
CDKN2C	Cyclin-dependent kinase inhibitor 2C (p18, inhibits CDK4)
LYL1	Lymphoblastic leukemia derived sequence 1
TCF3	Transcription factor 3 (E2A immunoglobulin enhancer binding factors E12/E47)
HHEX	Hematopoietically expressed homeobox
SIX1	SIX homeobox 1
HOXA11	Homeobox A11
PTCRA	Pre-T-cell antigen receptor alpha
MLLT1	Myeloid/lymphoid or mixed-lineage leukemia (trithorax homolog, *Drosophila*), translocated to 1
HOXA10	Homeobox A10

**Table 11:** Classification accuracies of 21 T-ALL related genes in KEGG pathways in MILE and independent microarray data by two different classifiers in WEKA.

Datasets	J48	RBF network
MILE study dataset	93.11	95.95
Independent dataset	84.21	86.84

**Table 12:** The genes common to subnetwork genes and the 100 most differentially expressed genes.

Gene symbol	Gene title	GO: function	GO: process
CLU	Clusterin	Protein binding	Antiapoptosis, apoptosis, cell death, complement activation, classical pathway, endocrine pancreas development, innate immune response, lipid metabolic process, neurite morphogenesis, positive regulation of cell differentiation, positive regulation of cell proliferation, and response to oxidative stress

ITGA2B	Integrin, alpha 2b (platelet glycoprotein IIb of IIb/IIIa complex, antigen CD41)	Calcium ion binding, identical protein binding, protein binding, and receptor activity	Cell adhesion, cell adhesion, and integrin-mediated signaling pathway

**Table 13:** The genes common to subnetwork genes and the 200 most differentially expressed genes.

Gene symbol	Gene title	GO: function	GO: process
ADD2	Adducin 2 (beta)	Actin binding, calmodulin binding, and metal-ion binding	No enrichments

CD36	CD36 molecule (thrombospondin receptor)	Lipoprotein binding, low-density lipoprotein receptor activity, and receptor activity	Blood coagulation, cell adhesion, lipid metabolic process, lipoprotein transport, and transport

CLU	Clusterin	Protein binding	Antiapoptosis, apoptosis, cell death, complement activation, classical pathway, endocrine pancreas development, innate immune response, lipid metabolic process, neurite morphogenesis, positive regulation of cell differentiation, positive regulation of cell proliferation, and response to oxidative stress

GSN	Gelsolin (amyloidosis, Finnish type)	Actin binding, calcium ion binding, and protein binding	Actin filament polymerization, actin filament severing, and barbed-end actin filament capping

ITGA2B	Integrin, alpha 2b (platelet glycoprotein IIb of IIb/IIIa complex, antigen CD41)	Calcium ion binding, identical protein binding, protein binding, and receptor activity	Cell adhesion, cell adhesion, and integrin-mediated signaling pathway

LTB4R	Leukotriene B4 receptor	Leukotriene B4 receptor activity, nucleotide binding, and receptor activity	G-protein signaling, coupled to IP3 second messenger (phospholipase C activating), cell motility, immune response, inflammatory response, muscle contraction, and signal transduction

TLR4	Toll-like receptor 4	Lipopolysaccharide binding, protein binding, and transmembrane receptor activity	I-kappaB kinase/NF-kappaB cascade, T-helper 1 type immune response, detection of fungus, inflammatory response, innate immune response, macrophage activation, negative regulation of osteoclast differentiation, positive regulation of interleukin-12 biosynthetic process, positive regulation of interleukin-12 biosynthetic process, positive regulation of interleukin-8 biosynthetic process, positive regulation of tumor necrosis factor biosynthetic process, and signal transduction

TUBB1	Tubulin, beta-1	GTP binding, GTPase activity, nucleotide binding, and structural molecule activity	Microtubule-based movement, protein polymerization

**Table 14:** The classification accuracies of the 100 and 200 most differential genes between T-ALL and healthy samples.

Genes	J48	RBF network
100 most DE	95	98
200 most DE	95	98
