# Supplementary material for: Identification of Interconnected Markers for T-Cell Acute Lymphoblastic Leukemia
Source: Biomed Res Int. 2013 Jul 15;2013:210253. doi: 10.1155/2013/210253 (PMC3727179; doi:10.1155/2013/210253)
Supplement: Supplementary file 1 — The Supplementary Material contains 21 tables. The genes in the subnetworks and their functions are listed in Tables S1 through S19 (for each individual subnetwork, there is one table). Tables S20 and S21 display titles and functions of the 100 and 200 most differentially expressed genes. [file 210253.f1.pdf]

SUPPLEMENTARY MATERIALS

The genes in the subnetworks and their functions are given as tables S1 through S19 (for each individual subnetwork, there is one table).

Table S1: The genes in Subnetwork-1 and their functions according to GO

Gene Symbol	Gene title	GO:Function	GO:Process
MYO10	myosin X	ATP binding, actin binding, motor activity, nucleotide binding	signal transduction
TNNI2	troponin I type 2 (skeletal, fast)	Contributes to actin binding, protein binding, troponin T binding	positive regulation of transcription, skeletal muscle contraction
KCNN4	potassium intermediate/small conductance calcium-activated channel, subfamily N, member 4	calcium-activated potassium channel activity, calmodulin binding, ion channel activity, voltage-gated potassium channel activity	defense response, ion transport, positive regulation of protein secretion, potassium ion transport, saliva secretion
ADD2	adducin 2 (beta)	actin binding, calmodulin binding, metal ion binding	
CALM1	calmodulin 1 (phosphorylase kinase, delta)		

Table S2: The genes in Subnetwork-2 and their functions according to GO

Gene Symbol	Gene title	GO:Function	GO:Process
CCR5	chemokine (C-C motif) receptor 5	C-C chemokine receptor activity, C-C chemokine receptor activity, actin binding, coreceptor activity, phosphoinositide phospholipase C activity, protein binding, receptor activity, rhodopsin-like receptor activity	G-protein coupled receptor protein signaling pathway, cell-cell signaling, cellular defense response, chemotaxis, elevation of cytosolic calcium ion concentration, immune response, inflammatory response, initiation of viral infection, signal transduction
CCL5	chemokine (C-C motif) ligand 5	chemoattractant activity, chemokine activity, signal transducer activity	cell adhesion, cell motility, cell-cell signaling, cellular calcium ion homeostasis, cellular defense response, chemotaxis, exocytosis, immune response, Inflammatory response, negative regulation of viral genome replication, response to oxidative stress, response to virus, signal transduction
PTK2	PTK2 protein tyrosine kinase 2	ATP binding, SH2 domain binding, non-membrane spanning protein tyrosine kinase activity, nucleotide binding, protein binding, protein tyrosine kinase activity, signal transducer activity, transferase activity	integrin-mediated signaling pathway, protein amino acid phosphorylation, signal complex assembly
ORM1	orosomucoid 1	protein binding	acute-phase response, inflammatory response
CCL14	chemokine (C-C motif) ligand 14	chemokine activity, protein binding, signal transducer activity	cellular calcium ion homeostasis, immune response, positive regulation of cell proliferation
ARL6IP5	ADP-ribosylation-like factor 6 interacting protein 5	protein binding	L-glutamate transport
ADRBK1	adrenergic, beta, receptor kinase 1	ATP binding, G-protein coupled receptor kinase activity, beta-adrenergic receptor kinase activity, nucleotide binding, protein kinase activity,protein serine/threonine kinase activity, receptor activity, signal transducer activity, transferase	cardiac muscle contraction, desensitization of G-protein coupled receptor protein signaling pathway, heart development, negative regulation of striated muscle contraction, negative regulation of the force of heart contraction by chemical signal, protein amino acid phosphorylation, regulation of the force of heart contraction, signal transduction, tachykinin signaling pathway

		activity	

Table S3: The genes in Subnetwork-3 and their functions according to GO

**Gene Symbol**	**Gene title**	**GO:Function**	**GO:Process**
CD36	CD36 molecule (thrombospondin receptor)	lipoprotein binding, low-density lipoprotein receptor activity, receptor activity	blood coagulation, cell adhesion, lipid metabolic process, lipoprotein transport, transport
ITGA2B	integrin, alpha 2b (platelet glycoprotein IIb of IIb/IIIa complex, antigen CD41)	calcium ion binding, identical protein binding, protein binding, receptor activity	cell adhesion, integrin-mediated signaling pathway
COL5A3	collagen, type V, alpha 3	extracellular matrix structural constituent, heparin binding	cell adhesion ,cell-matrix adhesion, muscle development, organ morphogenesis, phosphate transport
P4HB	procollagen-proline, 2-oxoglutarate 4-dioxygenase (proline 4-hydroxylase), beta polypeptide	isomerase activity, procollagen-proline 4-dioxygenase activity, protein binding, protein disulfide isomerase activity	cell redox homeostasis, peptidyl-proline hydroxylation to 4-hydroxy-L-proline
COL1A2	collagen, type I, alpha 2	extracellular matrix structural constituent, protein binding	phosphate transport, sensory perception of sound, skeletal development, transmembrane receptor protein tyrosine kinase signaling pathway

Table S4: The genes in Subnetwork-4 and their functions according to GO

Gene Symbol	Gene title	GO:Function	GO:Process
STRA13	stimulated by retinoic acid 13 homolog (mouse)	DNA binding	
SMAD4	SMAD family member 4	SMAD binding, protein homodimerization activity, sequence-specific DNA binding ,transcription activator activity, transcription factor activity	BMP signaling pathway, SMAD protein complex assembly, anterior/posterior pattern formation, kidney development, negative regulation of cell growth, negative regulation of cell proliferation, negative regulation of transcription, DNA-dependent, positive regulation of transcription from RNA polymerase II promoter, regulation of binding, regulation of transforming growth factor beta receptor signaling pathway, regulation of transforming growth factor-beta2 production, response to hypoxia, transcription, ureteric bud branching
UBE2I	ubiquitin-conjugating enzyme E2I (UBC9 homolog, yeast)	HLH domain binding, ligase activity ,protein binding, small conjugating protein ligase activity	cell cycle, cell division, chromosome segregation, mitosis, regulation of protein metabolic process, ubiquitin cycle, ubiquitin-dependent protein catabolic process
TP53	tumor protein p53	ATP binding, DNA strand annealing activity, chromatin binding, copper ion binding, enzyme binding, metal ion binding, nuclease activity, protein N-terminus binding, protein heterodimerization activity, transcription factor activity, zinc ion binding	DNA damage response, apoptosis, signal transduction by p53 class mediator resulting in induction of apoptosis, ER overload response, RNA-protein covalent cross-linking, base-excision repair, caspase activation via cytochrome c, cell aging, cell cycle arrest, cell differentiation, cell proliferation, cellular response to glucose starvation, induction of apoptosis by intracellular signals, multicellular organismal development, negative regulation of cell cycle, negative regulation of cell growth, negative regulation of helicase activity, positive regulation of transcription from RNA polymerase II promoter, protein complex assembly, protein localization, regulation of mitochondrial membrane permeability, regulation of transcription, response to tumor cell
RANBP2	RAN binding protein 2	Ran GTPase binding, isomerase activity, metal ion binding, peptidyl-prolyl cis-trans isomerase	intracellular protein transport across a membrane,I ntracellular transport, mRNA transport, protein folding, protein import into nucleus, ubiquitin cycle

		activity, protein binding, zinc ion binding	
ATF2	activating transcription factor 2	RNA polymerase II transcription factor activity, metal ion binding,p rotein dimerization activity, sequence-specific DNA binding, transcription coactivator activity, transcription factor activity, zinc ion binding	regulation of transcription
WT1	Wilms tumor 1	metal ion binding, transcription factor activity, zinc ion binding	negative regulation of cell cycle, negative regulation of transcription from RNA polymerase II promoter
SOX4	SRY (sex determining region Y)-box 4	transcription factor activity	regulation of transcription
TFAP2C	transcription factor AP-2 gamma (activating enhancer binding protein 2 gamma)	protein dimerization activity, transcription factor activity	cell differentiation, cell-cell signaling, regulation of transcription from RNA polymerase II promoter, transcription

Table S5: The genes in Subnetwork-5 and their functions according to GO

**Gene Symbol**	**Gene title**	**GO:Function**	**GO:Process**
GRK6	G protein-coupled receptor kinase 6	ATP binding, nucleotide binding, signal transducer activity, transferase activity	amino acid phosphorylation, regulation of G-protein coupled receptor protein signaling pathway, signal transduction
LTB4R	leukotriene B4 receptor	nucleotide binding, receptor activity	G-protein signaling, coupled to IP3 second messenger (phospholipase C activating), cell motility, immune response, inflammatory response, muscle contraction, signal transduction
SNCA	synuclein, alpha (non A4	identical protein binding	anti-apoptosis, central nervous system development, dopamine biosynthetic process, phospholipid metabolic

	component of amyloid precursor)		process, positive regulation of neurotransmitter secretion, regulation of locomotion, regulation of long-term neuronal synaptic plasticity, response to drug, synaptic transmission, dopaminergic, synaptic vesicle transport
TBXA2R	thromboxane A2 receptor	rhodopsin-like receptor activity	G-protein coupled receptor protein signaling pathway, signal transduction

Table S6: The genes in Subnetwork-6 and their functions according to GO

Gene Symbol	Gene title	GO:Function	GO:Process
ATXN3	ataxin 3	hydrolase activity ,protein binding	cell death, nervous system development, nucleotide-excision repair, regulation of transcription, DNA-dependent, synaptic transmission, transcription
SALL2	sal-like 2 (Drosophila)	metal ion binding, transcription factor activity, zinc ion binding	regulation of transcription
TSPAN3	tetraspanin 3		
PRTFDC1	phosphoribosyl transferase domain containing 1	hypoxanthine phosphoribosyltransferase activity	nucleoside metabolic process, purine ribonucleoside salvage
EWSR1	Ewing sarcoma breakpoint region 1	RNA binding, calmodulin binding, metal ion binding, nucleotide binding, sequence-specific DNA binding, transcription factor activity, zinc ion binding	regulation of transcription, DNA-dependent, transcription

Table S7: The genes in Subnetwork-7 and their functions according to GO

Gene Symbol	Gene title	GO:Function	GO:Process
PRTFDC1	phosphoribosyl transferase domain containing 1	hypoxanthine phosphoribosyltransferase activity	nucleoside metabolic process, purine ribonucleoside salvage
EWSR1	Ewing sarcoma breakpoint region	RNA binding, calmodulin binding, metal ion binding, nucleotide binding,	regulation of transcription, DNA-dependent,

	1	sequence-specific DNA binding, transcription factor activity, zinc ion binding	transcription
SSBP2	single-stranded DNA binding protein 2	single-stranded DNA binding, transcription regulator activity	regulation of transcription
CEACAM5	carcinoembryonic antigen-related cell adhesion molecule 5		
YWHAG	tyrosine 3-monooxygenase/tryptophan 5-monooxygenase activation protein, gamma polypeptide	insulin-like growth factor receptor binding, protein domain specific binding, protein kinase C binding, protein kinase C inhibitor activity	Negative regulation of protein kinase activity, protein targeting, regulation of neuron differentiation, regulation of signal transduction, regulation of synaptic plasticity

Table S8: The genes in Subnetwork-8 and their functions according to GO

**Gene Symbol**	**Gene title**	**GO:Function**	**GO:Process**
DVL1	dishevelled, dsh homolog 1 (Drosophila)	protein binding, signal transducer activity	Wnt receptor signaling pathway, anatomical structure morphogenesis, heart development, intracellular signaling cascade, multicellular organismal development
VANGL1	vang-like 1 (van gogh, Drosophila)	protein binding	multicellular organismal development
SMAD4	SMAD family member 4	SMAD binding, protein homodimerization activity, sequence-specific DNA binding, transcription activator activity, transcription factor activity	BMP signaling pathway, SMAD protein complex assembly, anterior/posterior pattern formation, kidney development, negative regulation of cell growth, negative regulation of cell proliferation, negative regulation of transcription, DNA-dependent, positive regulation of transcription from RNA polymerase II promoter, regulation of binding, regulation of transforming growth factor beta receptor signaling pathway, regulation of transforming growth factor-beta2 production, response to hypoxia, transcription, ureteric bud branching
USP13	ubiquitin specific peptidase 13 (isopeptidase T-3)	cysteine-type endopeptidase activity, metal ion binding, ubiquitin thiolesterase activity, zinc ion binding	ubiquitin cycle, ubiquitin-dependent protein catabolic process
VANGL2	vang-like 2 (van gogh, Drosophila)	protein binding	apical protein localization, establishment of planar polarity, heart looping, multicellular organismal development, neural tube closure, sensory cilium biogenesis

CXXC4	CXXC finger 4	DNA binding, PDZ domain binding, metal ion binding, zinc ion binding	negative regulation of Wnt receptor signaling pathway

Table S9: The genes in Subnetwork-9 and their functions according to GO

**Gene Symbol**	**Gene title**	**GO:Function**	**GO:Process**
GSN	gelsolin (amyloidosis, Finnish type)	actin binding, calcium ion binding, protein binding	actin filament polymerization, actin filament severing, barbed-end actin filament capping
TPM1	tropomyosin 1 (alpha)	actin binding, structural constituent of cytoskeleton, structural constituent of muscle	cell motility, regulation of heart contraction, regulation of muscle contraction
LASP1	LIM and SH3 protein1	SH3/SH2 adaptor activity, actin binding,ion transmembrane transporter activity, metal ion binding, zinc ion binding	cortical cytoskeleton organization and biogenesis, ion transport
TGM2	transglutaminase 2 (C polypeptide, protein-glutamine-gamma-glutamyltransferase)	GTP binding, acyltransferase activity, calcium ion binding, protein domain specific binding, protein-glutamine gamma-glutamyltransferase activity, transferase activity	induction of apoptosis, blood vessel remodeling, elevation of cytosolic calcium ion concentration during G-protein signaling, coupled to IP3 second messenger (phospholipase C activating), isopeptide cross-linking via N6-(L-isoglutamyl)-L-lysine, peptide cross-linking, positive regulation of I-kappaB kinase/NF-kappaB cascade, positive regulation of cell adhesion, positive regulation of inflammatory response, positive regulation of smooth muscle cell proliferation, protein homooligomerization
NCF1	neutrophil cytosolic factor 1, (chronic granulomatous disease,	GTP binding, GTPase activity, electron carrier activity, phosphoinositide	cell communication, cellular defense response

	autosomal1)	binding, protein binding	
ACTB	actin, beta	ATP binding, nucleotide binding, protein binding, structural constituent of cytoskeleton, structural molecule activity	cell motility, sensory perception of sound
P2RX7	purinergic receptor P2X, ligand-gated ion channel, 7	ATP binding, ATP-gated cation channel activity, ion channel activity	ion transport, signal transduction

Table S10: The genes in Subnetwork-10 and their functions according to GO

**Gene Symbol**	**Gene title**	**GO:Function**	**GO:Process**
TUBB1	tubulin, beta 1	GTP binding, GTPase activity, nucleotide binding, structural molecule activity	microtubule-based movement, protein polymerization

Table S11: The genes in Subnetwork-11 and their functions according to GO

Gene Symbol	Gene title	GO:Function	GO:Process
PLSCR4	phospholipid scramblase 4	calcium ion binding, phospholipid scramblase activity	blood coagulation,phospholipid scrambling
SGTB	small glutamine-rich tetratricopeptide repeat (TPR)-containing, beta	binding	
PLSCR1	phospholipid scramblase 1	calcium ion binding, phospholipid scramblase activity, protein binding	phospholipid scrambling,platelet activation,response to virus
GFI1B	growth factor independent 1B transcription repressor	DNA binding, metal ion binding, specific RNA polymerase II transcription factor activity, zinc ion binding	G1-specific transcription in mitotic cell cycle, cell proliferation, chromatin modification, multicellular organismal development, negative regulation of transcription from RNA polymerase II promoter, regulation of transcription
EFEMP2	EGF-containing fibulin-like extracellular matrix protein 2	calcium ion binding, extracellular matrix structural constituent, protein binding, transmembrane receptor activity	blood coagulation

Table S12: The genes in Subnetwork-12 and their functions according to GO

**Gene Symbol**	**Gene title**	**GO:Function**	**GO:Process**
ARL4C	ADP-ribosylation factor-like 4C	GTP binding, GTPase activity, nucleotide binding	small GTPase mediated signal transduction
UBQLN4	ubiquilin 4	identical protein binding	biological_process,protein modification process
STMN1	stathmin 1/oncoprotein 18	protein binding, signal transducer activity, tubulin binding	cell differentiation, intracellular signaling cascade, microtubule depolymerization, mitotic spindle organization and biogenesis, multicellular organismal development, nervous system development
IGFBP6	insulin-like growth factor binding protein 6	insulin-like growth factor binding	negative regulation of cell proliferation, regulation of cell growth, signal transduction
COPB1	coatomer protein complex, subunit beta 1	protein binding, structural molecule activity	COPI coating of Golgi vesicle, intra-Golgi vesicle-mediated transport, intracellular protein transport, membrane organization and biogenesis, retrograde vesicle-mediated transport, Golgi to ER, vesicle-mediated transport
CD99	CD99 molecule	protein binding	cell adhesion

Table S13: The genes in Subnetwork-13 and their functions according to GO

Gene Symbol	Gene title	GO:Function	GO:Process
NCR1	natural cytotoxicity triggering receptor 1	protein binding,receptor activity	cellular defense response, natural killer cell activation ,regulation of natural killer cell mediated cytotoxicity, signal transduction
C9	complement component 9		caspase activation, complement activation-alternative pathway ,complement activation-classical pathway, cytolysis, hemolysis by symbiont of host red blood cells, induction of apoptosis
CD59	CD59 molecule, complement regulatory protein	protein binding	blood coagulation, cell surface receptor linked signal transduction
CLU	clusterin	protein binding	anti-apoptosis, apoptosis, cell death, complement activation, classical pathway, endocrine pancreas development ,innate immune response, lipid metabolic process, neurite morphogenesis, positive regulation of cell differentiation, positive regulation of cell proliferation, response to oxidative stress
LEPR	leptin receptor	hematopoietin/interferon-class (D200-domain) cytokine receptor activity, protein binding	cell surface receptor linked signal transduction, energy reserve metabolic process, multicellular organismal development

APP	amyloid beta (A4) precursor protein (peptidase nexin-II, Alzheimer disease)	acetylcholine receptor binding, copper ion binding, heparin binding, iron ion binding, serine-type endopeptidase inhibitor activity, zinc ion binding	Notch signaling pathway, apoptosis, cell adhesion, cellular copper ion homeostasis, endocytosis, neuromuscular process

Table S14: The genes in Subnetwork-14 and their functions according to GO

**Gene Symbol**	**Gene title**	**GO:Function**	**GO:Process**
MEP1A	meprin A, alpha (PABA peptide hydrolase)	astacin activity, metal ion binding, metallopeptidase activity, zinc ion binding	digestion, proteolysis
SERPINI1	serpin peptidase inhibitor, clade I (neuroserpin), member 1	serine-type endopeptidase inhibitor activity	central nervous system development, peripheral nervous system development
THBS1	thrombospondin 1	calcium ion binding, endopeptidase inhibitor activity, heparin binding, protein binding, signal transducer activity, structural molecule activity	cell adhesion, multicellular organismal development
MMP9	matrix metallopeptidase 9 (gelatinase B, 92kDa gelatinase, 92kDa type IV collagenase)	calcium ion binding, collagen binding, collagenase activity, gelatinase B activity, zinc ion binding	collagen catabolic process, extracellular matrix organization and biogenesis, macrophage differentiation, metabolic process, positive regulation of apoptosis, proteolysis, skeletal development

CHGA	chromogranin A (parathyroid secretory protein 1)	calcium ion binding, protein binding	regulation of blood pressure
SPARC	secreted protein, acidic, cysteine-rich (osteonectin)	calcium ion binding, collagen binding, copper ion binding	ossification, transmembrane receptor protein tyrosine kinase signaling pathway
CLEC3B	C-type lectin domain family 3, member B	sugar binding	skeletal development
PLG	plasminogen	apolipoprotein binding, calcium ion binding, peptidase activity, plasmin activity	blood coagulation, induction of apoptosis, negative regulation of angiogenesis, negative regulation of blood vessel endothelial cell migration, negative regulation of cell proliferation, negative regulation of fibrinolysis, proteolysis, tissue remodeling

Table S15: The genes in Subnetwork-15 and their functions according to GO

**Gene Symbol**	**Gene title**	**GO:Function**	**GO:Process**
CAT	catalase	NADP binding, catalase activity, heme binding, iron ion binding, protein homodimerization activity	UV protection, hydrogen peroxide catabolic process, negative regulation of apoptosis, oxidation reduction, response to reactive oxygen species
TLR10	toll-like receptor 10	protein binding,transmembrane receptor activity	I-kappaB kinase/NF-kappaB cascade,inflammatory response,innate immune response,signal transduction
TLR1	toll-like receptor 1	protein binding, transmembrane receptor activity	inflammatory response, innate immune response, macrophage activation, positive regulation of interleukin-6 biosynthetic process, positive regulation of tumor necrosis factor biosynthetic process, signal transduction
TLR2	toll-like receptor 2	Gram-positive bacterial binding, lipopolysaccharide receptor activity, peptidoglycan binding, protein	induction of apoptosis, I-kappaB kinase/NF-kappaB cascade, MyD88-dependent toll-like receptor signaling

		binding, transmembrane receptor activity	pathway, cell surface pattern recognition receptor signaling pathway, chloramphenicol transport, inflammatory response, innate immune response, positive regulation of tumor necrosis factor production, response to molecule of fungal origin, signal transduction
FUT7	fucosyltransferase 7 (alpha (1,3) fucosyltransferase)	transferase activity, transferring glycosyl groups	L-fucose catabolic process, protein amino acid glycosylation

Table S16: The genes in Subnetwork-16 and their functions according to GO

**Gene Symbol**	**Gene title**	**GO:Function**	**GO:Process**
ABL1	c-abl oncogene 1, receptor tyrosine kinase	ATP binding,bDNA binding,bkinase activity,bmagnesium ion bindingb,manganese ion binding, membrane spanning protein tyrosine kinase activity, nucleotide binding, protein C-terminus binding, transferase activity	DNA damage response, signal transduction resulting in induction of apoptosis, S-phase-specific transcription in mitotic cell cycle, actin cytoskeleton organization and biogenesis, cell adhesion, mismatch repair, peptidyl-tyrosine phosphorylation, positive regulation of oxidoreductase activity, protein modification process, regulation of transcription
XRCC6	X-ray repair complementing defective repair in	ATP-dependent DNA helicase activity, DNA binding, double-stranded DNA binding, protein	DNA ligation, DNA repair, double-strand break repair via nonhomologous end joining, initiation of viral infection, positive regulation of transcription,

	Chinese hamster cells 6 (Ku autoantigen, 70kDa)	binding	DNA-dependent, provirus integration
GAL3ST4	galactose-3-O-sulfotransferase 4	3'-phosphoadenosine 5'-phosphosulfate binding ,proteoglycan sulfotransferase activity, transferase activity	biosynthetic process, cell-cell signaling, glycoprotein metabolic process, oligosaccharide metabolic process, proteoglycan biosynthetic process, sulfur metabolic process
OVGP1	oviductal glycoprotein 1, 120kDa (mucin 9, oviductin)	catalytic activity, cation binding, chitinase activity	carbohydrate metabolic process, chitin catabolic process, female pregnancy, single fertilization
RASA4	RAS p21 protein activator 4	GTPase activator activity, metal ion binding, zinc ion binding	intracellular signaling cascade, regulation of small GTPase mediated signal transduction
HN1L	hematological and neurological expressed 1-like	0	0
DNTT	deoxynucleotidyltransferase, terminal	DNA binding, DNA-directed DNA polymerase activity, catalytic activity, magnesium ion binding, transferase activity	DNA modification, DNA replication
ABCD4	ATP-binding cassette, sub-family D (ALD), member 4	ATP binding, ATPase activity, coupled to transmembrane movement of substances, nucleotide binding,transporter activity	transport
MAPK8	mitogen-activated protein kinase 8	ATP binding, JUN kinase activity, MAP kinase activity, nucleotide binding, protein binding, protein serine/threonine kinase activity, transferase activity	activation of pro-apoptotic gene products, JUN phosphorylation, cell motility, negative regulation of apoptosis, protein amino acid phosphorylation, response to UV, response to stress, signal transduction

Table S17: The genes in Subnetwork-17 and their functions according to GO

**Gene Symbol**	**Gene title**	**GO:Function**	**GO:Process**
DNAJC3	DnaJ (Hsp40) homolog, subfamily C, member 3	heat shock protein binding, protein kinase inhibitor activity	response to unfolded protein, response to virus
IRAK2	interleukin-1 receptor-associated kinase 2	ATP binding, NF-kappaB-inducing kinase activity, identical protein binding ,protein kinase activity	I-kappaB kinase/NF-kappaB cascade, inflammatory response, amino acid phosphorylation, signal transduction
BTK	Bruton agammaglobulinemi	ATP binding, kinase activity, metal ion binding, non-membrane spanning	calcium-mediated signaling, induction of apoptosis by extracellular signals,

	a tyrosine kinase	protein tyrosine kinase activity, nucleotide binding, protein tyrosine kinase activity, transferase activity, zinc ion binding	intracellular signaling cascade, mesoderm development, amino acid phosphorylation
TIRAP	toll-interleukin 1 receptor (TIR) domain containing adaptor protein	protein binding, transmembrane receptor activity	I-kappaB kinase/NF-kappaB cascade, inflammatory response, Innate immune response, signal transduction
IL1RL1	interleukin 1 receptor-like 1	interleukin-1 receptor activity, transmembrane receptor activity	innate immune response, signal transduction
TLR5	toll-like receptor 5	transmembrane receptor activity	inflammatory response, innate immune response, signal transduction
TLR4	toll-like receptor 4	lipopolysaccharide binding,transmembrane receptor activity	I-kappaB kinase/NF-kappaB cascade, T-helper 1 type immune response, detection of fungus, inflammatory response, innate immune response ,macrophage activation, negative regulation of osteoclast differentiation, positive regulation of interleukin-12 biosynthetic process, positive regulation of tumor necrosis factor biosynthetic process, signal transduction

Table S18: The genes in Subnetwork-18 and their functions according to GO

**Gene Symbol**	**Gene title**	**GO:Function**	**GO:Process**
USP20	ubiquitin specific peptidase 20	cysteine-type peptidase activity, metal ion binding, ubiquitin thiolesterase activity, zinc ion binding	protein deubiquitination, ubiquitin-dependent protein catabolic process
PRKCI	protein kinase C, iota	ATP binding, atypical protein kinase C activity, diacylglycerol binding, metal	Golgi vesicle budding ,actin filament organization, cell-cell junction assembly and maintenance, cytoskeleton organization and biogenesis,

		ion binding, nucleotide binding, phospholipid binding, transferase activity, zinc ion binding	establishment and/or maintenance of epithelial cell polarity ,establishment of apical/basal cell polarity ,eye photoreceptor cell development, intracellular signaling cascade, membrane organization and biogenesis, amino acid phosphorylation, protein targeting to membrane, secretion, vesicle-mediated transport
PARD3	par-3 partitioning defective 3 homolog (C. elegans)	protein binding	activation of protein kinase C activity, asymmetric cell division, axonogenesis, cell cycle, establishment and/or maintenance of cell polarity, protein complex assembly
PNMA1	paraneoplastic antigen MA1	protein binding	central nervous system development, spermatogenesis

Table S19: The genes in Subnetwork-18 and their functions according to GO

Gene Symbol	Gene title	GO:Function	GO:Process
HHIP	hedgehog interacting protein	catalytic activity, protein binding	dorsal/ventral pattern formation, lung development, negative regulation of smoothened signaling pathway, neuroblast proliferation, organ morphogenesis, regulation of fibroblast growth factor receptor signaling pathway, signal transduction

The following tables (**Error! Reference source not found.**, **Error! Reference source not found.**) represent the functions and titles of 100 and 200 most differentially expressed genes.

Table S20: The titles and functions of 100 most differentially expressed genes.

Gene symbol	Gene title	GO:Function	GO:Process

ACPP	acid phosphatase, prostate	acid phosphatase activity, hydrolase activity	
AGPAT9	1-acylglycerol-3-phosphate O-acyltransferase 9	1-acylglycerol-3-phosphate O-acyltransferase activity, acyltransferase activity, transferase activity	metabolic process, phospholipid biosynthetic process
ALDH1A1	aldehyde dehydrogenase 1 family, member A1	Ras GTPase activator activity, aldehyde dehydrogenase (NAD) activity, aldehyde dehydrogenase (NAD) activity, androgen binding, oxidoreductase activity, retinal dehydrogenase activity	aldehyde metabolic process, metabolic process, oxidation reduction
ALDH3A2	aldehyde dehydrogenase 3 family, member A2	3-chloroallyl aldehyde dehydrogenase activity, aldehyde dehydrogenase (NAD) activity, aldehyde dehydrogenase (NAD(P)+) activity, oxidoreductase activity	aldehyde metabolic process, central nervous system development, epidermis development, lipid metabolic process, oxidation reduction, peripheral nervous system development
ALDH3B1	aldehyde dehydrogenase 3 family, member B1	3-chloroallyl aldehyde dehydrogenase activity, aldehyde dehydrogenase (NAD(P)+) activity, oxidoreductase activity	alcohol metabolic process, aldehyde metabolic process, lipid metabolic process, metabolic process, oxidation reduction
ANK1	ankyrin 1, erythrocytic	cytoskeletal adaptor activity, enzyme binding, spectrin binding, structural constituent of cytoskeleton	cytoskeleton organization and biogenesis, exocytosis, maintenance of epithelial cell polarity, signal transduction
ANKRD18A	ankyrin repeat domain 18A		
ANKRD22	ankyrin repeat domain 22		
APOC1	apolipoprotein C-I	lipid transporter activity	cholesterol efflux, lipid metabolic process, lipid transport, lipoprotein metabolic process, phospholipid efflux
ARHGAP24	Rho GTPase activating protein 24	GTPase activator activity, protein binding	angiogenesis, cell differentiation, multicellular organismal development, signal transduction
ATP8B4	ATPase, class I, type 8B, member 4	ATP binding, ATPase activity, coupled to transmembrane movement of ions, phosphorylative mechanism, hydrolase activity, magnesium ion binding, nucleotide binding, phospholipid-translocating ATPase activity	phospholipid transport, transport
B4GALT5	UDP-Gal:betaGlcNAc	galactosyltransferase activity,	carbohydrate metabolic process

	beta 1, 4-galactosyltransferase, polypeptide 5	manganese ion binding, metal ion binding, transferase activity, transferring glycosyl groups	
BCL2L15	BCL2-like 15		apoptosis
C1orf38	chromosome 1 open reading frame 38		cell adhesion
C22orf25	chromosome 22 open reading frame 25		
C9orf122	chromosome 9 open reading frame 122		
CCNDBP1	cyclin D-type binding-protein 1	protein binding	cell cycle
CCPG1	cell cycle progression 1		cell cycle
CDC14B	CDC14 cell division cycle 14 homolog B (S. cerevisiae)	hydrolase activity, protein serine/threonine phosphatase activity, protein tyrosine phosphatase activity, protein tyrosine/serine/threonine phosphatase activity	cell division, protein amino acid dephosphorylation
CEACAM1	carcinoembryonic antigen-related cell adhesion molecule 1 (biliary glycoprotein)	molecular_function	angiogenesis, biological_process, cell migration, homophilic cell adhesion, integrin-mediated signaling pathway
CEACAM3	carcinoembryonic antigen-related cell adhesion molecule 3		
CEACAM4	carcinoembryonic antigen-related cell adhesion molecule 4		
CETP	cholesteryl ester transfer protein, plasma	cholesterol binding, cholesterol transporter activity, phosphatidylcholine binding, phospholipid transporter activity, triglyceride binding	acylglycerol homeostasis, cholesterol homeostasis, cholesterol metabolic process, high-density lipoprotein particle remodeling, lipid metabolic process, low-density lipoprotein particle remodeling, phosphatidylcholine metabolic process, phospholipid homeostasis, phospholipid transport, reverse cholesterol transport, steroid metabolic process, triacylglycerol metabolic process, very-low-density lipoprotein particle remodeling
CLEC4D	C-type lectin domain family 4, member D	binding, sugar binding	immune response
CLEC5A	C-type lectin domain family 5, member A	binding, binding, sugar binding	cellular defense response, immune response, signal transduction
CLU	clusterin	protein binding	anti-apoptosis, apoptosis, cell death, complement activation, classical pathway, endocrine pancreas development, innate immune response, lipid metabolic process, neurite morphogenesis, positive regulation

			of cell differentiation, positive regulation of cell proliferation, response to oxidative stress
CPM	carboxypeptidase M	carboxypeptidase A activity, metal ion binding, metallopeptidase activity, zinc ion binding	anatomical structure morphogenesis, proteolysis
CST7	cystatin F (leukocystatin)	cysteine protease inhibitor activity	immune response
DEFT1P	defensin, theta 1 pseudogene		
FABP4	fatty acid binding protein 4, adipocyte	fatty acid binding, protein binding, transcription repressor activity, transporter activity	cholesterol homeostasis, cytokine production, negative regulation of protein kinase activity, negative regulation of transcription, positive regulation of inflammatory response, transport
FBN2	fibrillin 2 (congenital contractural arachnodactyly)	binding, calcium ion binding, extracellular matrix structural constituent	anatomical structure morphogenesis, embryonic limb morphogenesis
FBXO9	F-box protein 9	ubiquitin-protein ligase activity	protein ubiquitination
FRMD3	FERM domain containing 3	binding, cytoskeletal protein binding	
GGTA1	glycoprotein, alpha-galactosyltransferase 1	transferase activity, transferring hexosyl groups	carbohydrate metabolic process
GPR177	G protein-coupled receptor 177	receptor activity, signal transducer activity	positive regulation of I-kappaB kinase/NF-kappaB cascade
GPX3	glutathione peroxidase 3 (plasma)	glutathione binding, glutathione peroxidase activity, oxidoreductase activity, selenium binding, transcription factor binding	glutathione metabolic process, hydrogen peroxide catabolic process, oxidation reduction, protein homotetramerization, response to lipid hydroperoxide, response to oxidative stress
GYG1	glycogenin 1	glycogenin glucosyltransferase activity, glycogenin glucosyltransferase activity, protein binding, transferase activity, transferase activity, transferring hexosyl groups	carbohydrate biosynthetic process, glycogen biosynthetic process
GYPB	glycophorin B (MNS blood group)		
HBQ1	hemoglobin, theta 1	heme binding, iron ion binding, metal ion binding, oxygen binding, oxygen transporter activity	oxygen transport, transport

HSPC159	galectin-related protein	sugar binding	
IGKV1D-13	immunoglobulin kappa variable 1D-13		
INHBA	inhibin, beta A	activin inhibitor activity, cytokine activity, follistatin binding, growth factor activity, hormone activity, identical protein binding	G1/S transition of mitotic cell cycle, cell cycle arrest, cell differentiation, cell surface receptor linked signal transduction, cell-cell signaling, defense response, growth, hemoglobin biosynthetic process, induction of apoptosis, negative regulation of B cell differentiation, negative regulation of cell cycle, negative regulation of cell growth, negative regulation of follicle-stimulating hormone secretion, negative regulation of interferon-gamma biosynthetic process, negative regulation of macrophage differentiation, negative regulation of phosphorylation, nervous system development, ovarian follicle development, positive regulation of erythrocyte differentiation, positive regulation of follicle-stimulating hormone secretion, positive regulation of transcription from RNA polymerase II promoter, regulation of activin receptor signaling pathway, response to external stimulus, skeletal development
ITGA2B	integrin, alpha 2b (platelet glycoprotein IIb of IIb/IIIa complex, antigen CD41)	calcium ion binding, identical protein binding, protein binding, receptor activity	cell adhesion, cell adhesion, integrin-mediated signaling pathway
KEL	Kell blood group, metallo-endopeptidase	endopeptidase activity, endothelin-converting enzyme activity, metal ion binding, metallopeptidase activity, neprilysin activity, protein binding, zinc ion binding	protein amino acid N-linked glycosylation, proteolysis, vasoconstriction
LILRA2	leukocyte immunoglobulin-like receptor, subfamily A (with TM domain), member 2	antigen binding, receptor activity, receptor activity	defense response, immune response, signal transduction
LIN7A	lin-7 homolog A (C. elegans)	protein binding	exocytosis, neurotransmitter secretion, protein complex assembly, protein transport, synaptic vesicle transport
LOC643072	hypothetical LOC643072		
LOC643332	similar to Nonsecretory ribonuclease precursor (Ribonuclease US)	nucleic acid binding, pancreatic ribonuclease activity	

	(Eosinophil-derived neurotoxin) (RNase UpI-2) (Ribonuclease 2) (RNase 2)		
LOC644538	hypothetical protein LOC644538		
LOC646576	hypothetical LOC646576		
LRP3	low density lipoprotein receptor-related protein 3	receptor activity	receptor-mediated endocytosis
LRRN4	leucine rich repeat neuronal 4	protein binding	
MFAP3L	microfibrillar-associated protein 3-like		
MFSD10	major facilitator superfamily domain containing 10	tetracycline transporter activity, transporter activity	apoptosis, transport
MGC13057	hypothetical protein MGC13057		
MGST1	microsomal glutathione S-transferase 1	glutathione transferase activity, transferase activity	glutathione metabolic process
MYL4	myosin, light chain 4, alkali; atrial, embryonic	actin filament binding, actin monomer binding, calcium ion binding, motor activity, myosin II heavy chain binding, structural constituent of muscle	cardiac muscle contraction, muscle development, positive regulation of ATPase activity, regulation of the force of heart contraction, striated muscle contraction
NAALADL1	N-acetylated alpha-linked acidic dipeptidase-like 1	carboxypeptidase activity, dipeptidase activity, glutamate carboxypeptidase II activity, metal ion binding, metallopeptidase activity, zinc ion binding	proteolysis
NADK	NAD kinase	NAD+ kinase activity, NAD+ kinase activity, NAD+ kinase activity, metal ion binding, protein binding, transferase activity	ATP metabolic process, NAD metabolic process, metabolic process, phosphorylation
NFIA	nuclear factor I/A	transcription factor activity, transcription factor activity, transcription factor binding	DNA replication, regulation of transcription, DNA-dependent, regulation of transcription, DNA-dependent, transcription, viral genome replication
NKD2	naked cuticle homolog 2 (Drosophila)	calcium ion binding, protein binding	Wnt receptor signaling pathway, exocytosis, transport
NP	nucleoside phosphorylase	purine-nucleoside phosphorylase activity, purine-nucleoside phosphorylase	DNA modification, inosine catabolic process, nucleobase, nucleoside, nucleotide and nucleic acid metabolic

		activity, purine-nucleoside phosphorylase activity	process, nucleobase, nucleoside, nucleotide and nucleic acid metabolic process, positive regulation of T cell proliferation, positive regulation of alpha-beta T cell differentiation, urate biosynthetic process
NRGN	neurogranin (protein kinase C substrate, RC3)	calmodulin binding	nervous system development, signal transduction
NTN2L	netrin 2-like (chicken)	molecular_function, receptor binding	axon guidance
ORM2	orosomucoid 2	binding	acute-phase response
P2RY2	purinergic receptor P2Y, G-protein coupled, 2	purinergic nucleotide receptor activity, G-protein coupled, receptor activity, rhodopsin-like receptor activity	G-protein signaling, coupled to IP3 second messenger (phospholipase C activating), cellular ion homeostasis, signal transduction
PBX1	pre-B-cell leukemia homeobox 1	protein binding, sequence-specific DNA binding, transcription factor activity	C21-steroid hormone biosynthetic process, cell differentiation, regulation of transcription, DNA-dependent, sex determination
PDXK	pyridoxal (pyridoxine, vitamin B6) kinase	ATP binding, lithium ion binding, magnesium ion binding, nucleotide binding, potassium ion binding, protein homodimerization activity, pyridoxal kinase activity, pyridoxal phosphate binding, sodium ion binding, transferase activity, zinc ion binding	cell proliferation, pyridoxal phosphate biosynthetic process, pyridoxine biosynthetic process, vitamin B6 metabolic process
PF4	platelet factor 4 (chemokine (C-X-C motif) ligand 4)	chemokine activity, heparin binding	cytokine and chemokine mediated signaling pathway, immune response, leukocyte chemotaxis, negative regulation of angiogenesis, negative regulation of megakaryocyte differentiation, platelet activation
PLD1	phospholipase D1, phosphatidylcholine-specific	hydrolase activity, phosphoinositide binding, phospholipase D activity, protein binding	Ras protein signal transduction, cell communication, chemotaxis, lipid catabolic process, metabolic process
PPBP	pro-platelet basic protein (chemokine (C-X-C motif) ligand 7)	chemokine activity, glucose transmembrane transporter activity, growth factor activity	cell proliferation, chemotaxis, defense response to bacterium, glucose transport, immune response
PSTPIP1	proline-serine-threonine phosphatase interacting protein 1	catalytic activity, protein binding	cell adhesion, signal transduction

PTX3	pentraxin-related gene, rapidly induced by IL-1 beta	zymosan binding	inflammatory response, opsonization, positive regulation of nitric oxide biosynthetic process, positive regulation of phagocytosis, response to yeast
PYGL	phosphorylase, glycogen; liver (Hers disease, glycogen storage disease type VI)	AMP binding, ATP binding, bile acid binding, drug binding, glucose binding, glycogen phosphorylase activity, nucleotide binding, phosphorylase activity, protein homodimerization activity, purine binding, pyridoxal phosphate binding, transferase activity, transferring glycosyl groups	carbohydrate metabolic process, glucose homeostasis, glycogen metabolic process
QPCT	glutaminyl-peptide cyclotransferase (glutaminyl cyclase)	acyltransferase activity, glutaminyl-peptide cyclotransferase activity, metal ion binding, peptidase activity, transferase activity, zinc ion binding	protein modification process, proteolysis
RAB27A	RAB27A, member RAS oncogene family	GTP binding, GTPase activity, nucleotide binding	protein transport, small GTPase mediated signal transduction
REPS2	RALBP1 associated Eps domain containing 2	calcium ion binding, calcium ion binding, protein binding	epidermal growth factor receptor signaling pathway, protein complex assembly
RHD	Rh blood group, D antigen	molecular_function	biological_process
ROGDI	rogdi homolog (Drosophila)		
RXRA	retinoid X receptor, alpha	ligand-regulated transcription factor activity, metal ion binding, molecular_function, protein binding, retinoid-X receptor activity, sequence-specific DNA binding, steroid binding, steroid hormone receptor activity, tRNA-pseudouridine synthase activity, transcription coactivator activity, transcription factor activity, zinc ion binding	biological_process, positive regulation of transcription from RNA polymerase II promoter, regulation of transcription, DNA-dependent, signal transduction, transcription, vitamin metabolic process
SERPINB10	serpin peptidase inhibitor, clade B (ovalbumin), member 10	protein binding, serine-type endopeptidase inhibitor activity	
SIRPA	signal-regulatory protein alpha		cell adhesion

SIRPB1	signal-regulatory protein beta 1		cell surface receptor linked signal transduction
SLC22A15	solute carrier family 22, member 15	transporter activity	transport
SNTB1	syntrophin, beta 1 (dystrophin-associated protein A1, 59kDa, basic component 1)	actin binding, calcium ion binding, calmodulin binding	muscle contraction
SYNE1	spectrin repeat containing, nuclear envelope 1	actin binding, actin binding, lamin binding, structural molecule activity	Golgi organization and biogenesis, muscle cell differentiation, nuclear organization and biogenesis
TMEM50B	transmembrane protein 50B	molecular_function	biological_process
TMEM56	transmembrane protein 56		
TPRG1L	tumor protein p63 regulated 1-like		
TRIM10	tripartite motif-containing 10	metal ion binding, protein binding, zinc ion binding, zinc ion binding	hemopoiesis
TSPAN7	tetraspanin 7		protein amino acid N-linked glycosylation
UNQ464	GLPG464		
WIPI1	WD repeat domain, phosphoinositide interacting 1	androgen receptor binding, estrogen receptor binding, phosphatidylinositol 3-phosphate binding, receptor binding	autophagy, vesicle targeting, trans-Golgi to endosome

Table S21: The titles and functions of 200 most differentially expressed genes.

Gene symbol	Gene title	GO:Function	GO:Process
ABHD5	abhydrolase domain containing 5	aminopeptidase activity, hydrolase activity, protein binding	lipid metabolic process, proteolysis, sensory perception of sound
ACPP	acid phosphatase, prostate	acid phosphatase activity, hydrolase activity	
ACRBP	acrosin binding protein	molecular_function	biological_process
ADD2	adducin 2 (beta)	actin binding, calmodulin binding, metal ion binding	
AGPAT9	1-acylglycerol-3-phosphate O-acyltransferase 9	1-acylglycerol-3-phosphate O-acyltransferase activity, acyltransferase activity, transferase activity	metabolic process, phospholipid biosynthetic process
AGTRAP	angiotensin II receptor-associated protein		
ALAD	aminolevulinate, delta-, dehydratase	lyase activity, metal ion binding, porphobilinogen synthase activity, porphobilinogen synthase activity, zinc ion binding	heme biosynthetic process, metabolic process, porphyrin biosynthetic process
ALDH1A1	aldehyde dehydrogenase 1 family, member A1	Ras GTPase activator activity, aldehyde dehydrogenase (NAD) activity, aldehyde dehydrogenase (NAD) activity, androgen binding, oxidoreductase activity, retinal	aldehyde metabolic process, metabolic process, oxidation reduction

		dehydrogenase activity	
ALDH3A2	aldehyde dehydrogenase 3 family, member A2	3-chloroallyl aldehyde dehydrogenase activity, aldehyde dehydrogenase (NAD) activity, aldehyde dehydrogenase (NAD(P)+) activity, oxidoreductase activity	aldehyde metabolic process, central nervous system development, epidermis development, lipid metabolic process, oxidation reduction, peripheral nervous system development
ALDH3B1	aldehyde dehydrogenase 3 family, member B1	3-chloroallyl aldehyde dehydrogenase activity, aldehyde dehydrogenase [NAD(P)+] activity, oxidoreductase activity	alcohol metabolic process, aldehyde metabolic process, lipid metabolic process, metabolic process, oxidation reduction
ANK1	ankyrin 1, erythrocytic	cytoskeletal adaptor activity, enzyme binding, spectrin binding, structural constituent of cytoskeleton	cytoskeleton organization and biogenesis, exocytosis, maintenance of epithelial cell polarity, signal transduction
ANKRD18A	ankyrin repeat domain 18A		
ANKRD22	ankyrin repeat domain 22		
APOC1	apolipoprotein C-I	lipid transporter activity	cholesterol efflux, lipid metabolic process, lipid transport, lipoprotein metabolic process, phospholipid efflux
ARHGAP24	Rho GTPase activating protein 24	GTPase activator activity, protein binding	angiogenesis, cell differentiation, multicellular organismal development, signal transduction
ATP8B4	ATPase, class I, type 8B, member 4	ATP binding, ATPase activity, coupled to transmembrane movement of ions, phosphorylative mechanism, hydrolase activity, magnesium ion binding, nucleotide binding, phospholipid-translocating ATPase activity	phospholipid transport, transport
B3GALNT1	beta-1,3-N-acetylgalactosaminyltransferase 1 (globoside blood group)	UDP-galactose:beta-N-acetylglucosamine beta-1,3-galactosyltransferase activity, galactosylgalactosylglucosylceramide beta-D-acetylgalactosaminyltransferase activity, galactosyltransferase activity, magnesium ion binding, transferase activity, transferring glycosyl groups	oligosaccharide biosynthetic process, protein amino acid glycosylation
B4GALT5	UDP-Gal:betaGlcNAc beta 1,4-	galactosyltransferase activity, manganese ion binding, metal	carbohydrate metabolic process

	galactosyltransferase, polypeptide 5	ion binding, transferase activity, transferring glycosyl groups	
BCL2L15	BCL2-like 15		apoptosis
C10orf11	chromosome 10 open reading frame 11	protein binding	
C12orf59	chromosome 12 open reading frame 59		
C1orf38	chromosome 1 open reading frame 38		cell adhesion
C20orf27	chromosome 20 open reading frame 27		
C22orf25	chromosome 22 open reading frame 25		
C5orf4	chromosome 5 open reading frame 4	catalytic activity	metabolic process
C9orf122	chromosome 9 open reading frame 122		
CCDC109A	coiled-coil domain containing 109A		
CCNDBP1	cyclin D-type binding-protein 1	protein binding	cell cycle
CCPG1	cell cycle progression 1		cell cycle
CD36	CD36 molecule (thrombospondin receptor)	lipoprotein binding, low-density lipoprotein receptor activity, receptor activity	blood coagulation, cell adhesion, lipid metabolic process, lipoprotein transport, transport
CD3G	CD3g molecule, gamma (CD3-TCR complex)	T cell receptor binding, protein heterodimerization activity, receptor signaling complex scaffold activity, transmembrane receptor activity	T cell activation, cell surface receptor linked signal transduction, establishment and/or maintenance of cell polarity, protein complex assembly, protein transport, regulation of apoptosis
CDC14B	CDC14 cell division cycle 14 homolog B (S. cerevisiae)	hydrolase activity, protein serine/threonine phosphatase activity, protein tyrosine phosphatase activity, protein tyrosine/serine/threonine phosphatase activity	cell division, protein amino acid dephosphorylation
CEACAM1	carcinoembryonic antigen-related cell adhesion molecule 1 (biliary glycoprotein)	molecular_function	angiogenesis, biological_process, cell migration, homophilic cell adhesion, integrin-mediated signaling pathway
CEACAM3	carcinoembryonic antigen-related cell adhesion molecule 3		
CEACAM4	carcinoembryonic antigen-related cell adhesion molecule 4		

CEBPE	CCAAT/enhancer binding protein (C/EBP), epsilon	protein dimerization activity, sequence-specific DNA binding, transcription factor activity	cytokine biosynthetic process, defense response to bacterium, macrophage differentiation, phagocytosis, regulation of transcription, DNA-dependent, transcription
CES1	carboxylesterase 1 (monocyte/macrophage serine esterase 1)	carboxylesterase activity, carboxylesterase activity, hydrolase activity	metabolic process, response to toxin
CETP	cholesteryl ester transfer protein, plasma	cholesterol binding, cholesterol transporter activity, phosphatidylcholine binding, phospholipid transporter activity, triglyceride binding	acylglycerol homeostasis, cholesterol homeostasis, cholesterol metabolic process, high-density lipoprotein particle remodeling, lipid metabolic process, low-density lipoprotein particle remodeling, phosphatidylcholine metabolic process, phospholipid homeostasis, phospholipid transport, reverse cholesterol transport, steroid metabolic process, triacylglycerol metabolic process, very-low-density lipoprotein particle remodeling
CLEC1B	C-type lectin domain family 1, member B	binding, sugar binding, transmembrane receptor activity	cell surface receptor linked signal transduction, defense response
CLEC4D	C-type lectin domain family 4, member D	binding, sugar binding	immune response
CLEC5A	C-type lectin domain family 5, member A	binding, binding, sugar binding	cellular defense response, immune response, signal transduction
CLTCL1	clathrin, heavy chain-like 1	protein binding, signal transducer activity, structural molecule activity	anatomical structure morphogenesis, intracellular protein transport, receptor-mediated endocytosis, vesicle-mediated transport
CLU	clusterin	protein binding	anti-apoptosis, apoptosis, cell death, complement activation, classical pathway, endocrine pancreas development, innate immune response, lipid metabolic process, neurite morphogenesis, positive regulation of cell differentiation, positive regulation of cell proliferation, response to oxidative stress
COL17A1	collagen, type XVII, alpha 1	structural molecule activity	cell-matrix adhesion, epidermis development, phosphate transport
COQ2	coenzyme Q2 homolog,	prenyltransferase activity,	biosynthetic process, glycerol metabolic process, isoprenoid

	prenyltransferase (yeast)	transferase activity	biosynthetic process, ubiquinone biosynthetic process
CPM	carboxypeptidase M	carboxypeptidase A activity, metal ion binding, metallopeptidase activity, zinc ion binding	anatomical structure morphogenesis, proteolysis
CST7	cystatin F (leukocystatin)	cysteine protease inhibitor activity	immune response
CTSE	cathepsin E	cathepsin E activity, pepsin A activity, peptidase activity	antigen processing and presentation of exogenous peptide antigen via MHC class II, digestion, proteolysis
CTTN	cortactin	protein binding	
CYBB	cytochrome b-245, beta polypeptide (chronic granulomatous disease)	FAD binding, electron carrier activity, iron ion binding, metal ion binding, voltage-gated ion channel activity	inflammatory response, ion transport, oxidation reduction
DAPK2	death-associated protein kinase 2	ATP binding, ATP binding, calmodulin binding, calmodulin-dependent protein kinase activity, identical protein binding, nucleotide binding, protein kinase activity, transferase activity	apoptosis, induction of apoptosis, protein amino acid phosphorylation, protein amino acid phosphorylation, protein kinase cascade
DDEF1	development and differentiation enhancing factor 1	ARF GTPase activator activity, metal ion binding, protein binding, zinc ion binding	regulation of ARF GTPase activity
DEFT1P	defensin, theta 1 pseudogene		
DHRS9	dehydrogenase/reductase (SDR family) member 9	3-alpha(17-beta)-hydroxysteroid dehydrogenase (NAD+) activity, 3-alpha(17-beta)-hydroxysteroid dehydrogenase (NAD+) activity, alcohol dehydrogenase activity, alcohol dehydrogenase activity, binding, oxidoreductase activity, racemase and epimerase activity, retinol dehydrogenase activity, retinol dehydrogenase activity	9-cis-retinoic acid biosynthetic process, 9-cis-retinoic acid biosynthetic process, androgen metabolic process, androgen metabolic process, epithelial cell differentiation, oxidation reduction, progesterone metabolic process, retinol metabolic process
ENTPD1	ectonucleoside triphosphate diphosphohydrolase 1	calcium ion binding, hydrolase activity, magnesium ion binding	blood coagulation, cell adhesion
EPOR	erythropoietin receptor	erythropoietin receptor activity, guanyl-nucleotide exchange factor activity, protein binding	brain development, elevation of cytosolic calcium ion concentration, heart development, signal transduction, signal transduction,

			small GTPase mediated signal transduction
FABP4	fatty acid binding protein 4, adipocyte	fatty acid binding, protein binding, transcription repressor activity, transporter activity	cholesterol homeostasis, cytokine production, negative regulation of protein kinase activity, negative regulation of transcription, positive regulation of inflammatory response, transport
FBN2	fibrillin 2 (congenital contractural arachnodactyly)	binding, calcium ion binding, extracellular matrix structural constituent	anatomical structure morphogenesis, embryonic limb morphogenesis
FBXO9	F-box protein 9	ubiquitin-protein ligase activity	protein ubiquitination
FCAR	Fc fragment of IgA, receptor for	IgA binding, receptor activity	immune response
FCGR2C	Fc fragment of IgG, low affinity IIc, receptor for (CD32)	IgG binding, protein binding, receptor activity, transmembrane receptor activity	immune response
FGF13	fibroblast growth factor 13	growth factor activity, protein binding, protein kinase activator activity	MAPKKK cascade, cell-cell signaling, nervous system development, signal transduction
FLOT2	flotillin 2	protein binding	cell adhesion, epidermis development
FPR2	formyl peptide receptor 2	N-formyl peptide receptor activity, receptor activity, rhodopsin-like receptor activity	G-protein coupled receptor protein signaling pathway, cell adhesion, cell motility, chemotaxis, inflammatory response, signal transduction
FRMD3	FERM domain containing 3	binding, cytoskeletal protein binding	
FUT1	fucosyltransferase 1 (galactoside 2-alpha-L-fucosyltransferase, H blood group)	fucosyltransferase activity, galactoside 2-alpha-L-fucosyltransferase activity, transferase activity, transferring glycosyl groups	L-fucose catabolic process, carbohydrate metabolic process, protein amino acid glycosylation
GATA1	GATA binding protein 1 (globin transcription factor 1)	metal ion binding, sequence-specific DNA binding, transcription factor activity, zinc ion binding	multicellular organismal development, regulation of transcription from RNA polymerase II promoter, transcription
GGTA1	glycoprotein, alpha-galactosyltransferase 1	transferase activity, transferring hexosyl groups	carbohydrate metabolic process
GNG11	guanine nucleotide binding protein (G protein), gamma 11	GTPase activity, signal transducer activity	G-protein coupled receptor protein signaling pathway, hormone-mediated signaling, signal transduction

GPR177	G protein-coupled receptor 177	receptor activity, signal transducer activity	positive regulation of I-kappaB kinase/NF-kappaB cascade
GPR84	G protein-coupled receptor 84	receptor activity, rhodopsin-like receptor activity	G-protein coupled receptor protein signaling pathway, biological_process, signal transduction
GPX3	glutathione peroxidase 3 (plasma)	glutathione binding, glutathione peroxidase activity, oxidoreductase activity, selenium binding, transcription factor binding	glutathione metabolic process, hydrogen peroxide catabolic process, oxidation reduction, protein homotetramerization, response to lipid hydroperoxide, response to oxidative stress
GSN	gelsolin (amyloidosis, Finnish type)	actin binding, calcium ion binding, protein binding	actin filament polymerization, actin filament severing, barbed-end actin filament capping
GYG1	glycogenin 1	glycogenin glucosyltransferase activity, glycogenin glucosyltransferase activity, protein binding, transferase activity, transferase activity, transferring hexosyl groups	carbohydrate biosynthetic process, glycogen biosynthetic process
GYPB	glycophorin B (MNS blood group)		
HBQ1	hemoglobin, theta 1	heme binding, iron ion binding, metal ion binding, oxygen binding, oxygen transporter activity	oxygen transport, transport
HLX	H2.0-like homeobox	sequence-specific DNA binding, sequence-specific DNA binding, transcription factor activity	cell differentiation, multicellular organismal development, regulation of transcription, DNA-dependent
HPR	haptoglobin-related protein	catalytic activity, hemoglobin binding, serine-type endopeptidase activity	proteolysis
HPSE	heparanase	beta-glucuronidase activity, calcium ion binding, hydrolase activity, magnesium ion binding	proteoglycan metabolic process
HSPC159	galectin-related protein	sugar binding	
HTRA3	HtrA serine peptidase 3	insulin-like growth factor binding, peptidase activity, protein binding, serine-type endopeptidase activity	proteolysis, regulation of cell growth
IGKV1D-13	immunoglobulin kappa variable 1D-13		
IGKV1OR15-	immunoglobulin kappa variable 1/OR15-118		

118			
IMPACT	Impact homolog (mouse)	molecular_function	biological_process, regulation of translation
INHBA	inhibin, beta A	activin inhibitor activity, cytokine activity, follistatin binding, growth factor activity, hormone activity, identical protein binding	G1/S transition of mitotic cell cycle, cell cycle arrest, cell differentiation, cell surface receptor linked signal transduction, cell-cell signaling, defense response, growth, hemoglobin biosynthetic process, induction of apoptosis, negative regulation of B cell differentiation, negative regulation of cell cycle, negative regulation of cell growth, negative regulation of follicle-stimulating hormone secretion, negative regulation of interferon-gamma biosynthetic process, negative regulation of macrophage differentiation, negative regulation of phosphorylation, nervous system development, ovarian follicle development, positive regulation of erythrocyte differentiation, positive regulation of follicle-stimulating hormone secretion, positive regulation of transcription from RNA polymerase II promoter, regulation of activin receptor signaling pathway, response to external stimulus, skeletal development
IRX5	iroquois homeobox 5	sequence-specific DNA binding, transcription factor activity	cell development, regulation of transcription, DNA-dependent
ITGA2B	integrin, alpha 2b (platelet glycoprotein IIb of IIb/IIIa complex, antigen CD41)	calcium ion binding, identical protein binding, protein binding, receptor activity	cell adhesion, cell adhesion, integrin-mediated signaling pathway
KEL	Kell blood group, metallo-endopeptidase	endopeptidase activity, endothelin-converting enzyme activity, metal ion binding, metallopeptidase activity, neprilysin activity, protein binding, zinc ion binding	protein amino acid N-linked glycosylation, proteolysis, vasoconstriction
KLF1	Kruppel-like factor 1 (erythroid)	metal ion binding, transcription factor activity, zinc ion binding	chromatin remodeling, erythrocyte maturation, regulation of transcription, DNA-dependent, transcription
LILRA2	leukocyte immunoglobulin-like	antigen binding, receptor	defense response, immune response,

	receptor, subfamily A (with TM domain), member 2	activity, receptor activity	signal transduction
LIN7A	lin-7 homolog A (C. elegans)	protein binding	exocytosis, neurotransmitter secretion, protein complex assembly, protein transport, synaptic vesicle transport
LOC388588	hypothetical LOC388588		
LOC643072	hypothetical LOC643072		
LOC643332	similar to Nonsecretory ribonuclease precursor (Ribonuclease US) (Eosinophil-derived neurotoxin) (RNase UpI-2) (Ribonuclease 2) (RNase 2)	nucleic acid binding, pancreatic ribonuclease activity	
LOC644538	hypothetical protein LOC644538		
LOC646576	hypothetical LOC646576		
LOXL3	lysyl oxidase-like 3	copper ion binding, copper ion binding, metal ion binding, oxidoreductase activity, protein-lysine 6-oxidase activity, protein-lysine 6-oxidase activity, scavenger receptor activity	biological_process, oxidation reduction
LRG1	leucine-rich alpha-2-glycoprotein 1	molecular_function, protein binding	biological_process
LRP3	low density lipoprotein receptor-related protein 3	receptor activity	receptor-mediated endocytosis
LRRN4	leucine rich repeat neuronal 4	protein binding	
LTB4R	leukotriene B4 receptor	leukotriene B4 receptor activity, nucleotide binding, receptor activity	G-protein signaling, coupled to IP3 second messenger (phospholipase C activating), cell motility, immune response, inflammatory response, muscle contraction, signal transduction
MANSC1	MANSC domain containing 1	chitinase activity	
MFAP3L	microfibrillar-associated protein 3-like		
MFAP3L	microfibrillar-associated protein 3-like		

MFSD10	major facilitator superfamily domain containing 10	tetracycline transporter activity, transporter activity	apoptosis, transport
MGC13057	hypothetical protein MGC13057		
MGST1	microsomal glutathione S-transferase 1	glutathione transferase activity, transferase activity	glutathione metabolic process
MOSC1	MOCO sulphurase C-terminal domain containing 1	molybdenum ion binding, oxidoreductase activity, pyridoxal phosphate binding	oxidation reduction
MYL4	myosin, light chain 4, alkali; atrial, embryonic	actin filament binding, actin monomer binding, calcium ion binding, motor activity, myosin II heavy chain binding, structural constituent of muscle	cardiac muscle contraction, muscle development, positive regulation of ATPase activity, regulation of the force of heart contraction, striated muscle contraction
NAALADL1	N-acetylated alpha-linked acidic dipeptidase-like 1	carboxypeptidase activity, dipeptidase activity, glutamate carboxypeptidase II activity, metal ion binding, metallopeptidase activity, zinc ion binding	proteolysis
NADK	NAD kinase	NAD+ kinase activity, NAD+ kinase activity, NAD+ kinase activity, metal ion binding, protein binding, transferase activity	ATP metabolic process, NAD metabolic process, metabolic process, phosphorylation
NFIA	nuclear factor I/A	transcription factor activity, transcription factor activity, transcription factor binding	DNA replication, regulation of transcription, DNA-dependent, regulation of transcription, DNA-dependent, transcription, viral genome replication
NKD2	naked cuticle homolog 2 (Drosophila)	calcium ion binding, protein binding	Wnt receptor signaling pathway, exocytosis, transport
NP	nucleoside phosphorylase	purine-nucleoside phosphorylase activity, purine-nucleoside phosphorylase activity, purine-nucleoside phosphorylase activity	DNA modification, inosine catabolic process, nucleobase, nucleoside, nucleotide and nucleic acid metabolic process, nucleobase, nucleoside, nucleotide and nucleic acid metabolic process, positive regulation of T cell proliferation, positive regulation of alpha-beta T cell differentiation, urate biosynthetic process
NPL	N-acetylneuraminate pyruvate lyase (dihydrodipicolinate	N-acetylneuraminate lyase activity, lyase activity	carbohydrate metabolic process, metabolic process

	synthase)		
NRGN	neurogranin (protein kinase C substrate, RC3)	calmodulin binding	nervous system development, signal transduction
NTN2L	netrin 2-like (chicken)	molecular_function, receptor binding	axon guidance
NUDT16P	nudix (nucleoside diphosphate linked moiety X)-type motif 16 pseudogene		
OLR1	oxidized low density lipoprotein (lectin-like) receptor 1	low-density lipoprotein receptor activity, protein binding, receptor activity, sugar binding	blood circulation, cell adhesion, immune response, inflammatory response, proteolysis
ORM2	orosomucoid 2	binding	acute-phase response
P2RY2	purinergic receptor P2Y, G-protein coupled, 2	purinergic nucleotide receptor activity, G-protein coupled, receptor activity, rhodopsin-like receptor activity	G-protein signaling, coupled to IP3 second messenger (phospholipase C activating), cellular ion homeostasis, signal transduction
PADI2	peptidyl arginine deiminase, type II	calcium ion binding, hydrolase activity, protein-arginine deiminase activity	peptidyl-citrulline biosynthetic process from peptidyl-arginine, protein modification process
PADI4	peptidyl arginine deiminase, type IV	calcium ion binding, hydrolase activity, protein-arginine deiminase activity, protein-arginine deiminase activity	chromatin modification, peptidyl-citrulline biosynthetic process from peptidyl-arginine, protein modification process, protein modification process, regulation of transcription, DNA-dependent, transcription
PARVB	parvin, beta	actin binding, protein binding	cell adhesion
PBX1	pre-B-cell leukemia homeobox 1	protein binding, sequence-specific DNA binding, transcription factor activity	C21-steroid hormone biosynthetic process, cell differentiation, regulation of transcription, DNA-dependent, sex determination
PCOLCE2	procollagen C-endopeptidase enhancer 2	heparin binding, protein binding	
PDXK	pyridoxal (pyridoxine, vitamin B6) kinase	ATP binding, lithium ion binding, magnesium ion binding, nucleotide binding, potassium ion binding, protein homodimerization activity, pyridoxal kinase activity, pyridoxal phosphate binding, sodium ion binding, transferase activity, zinc ion binding	cell proliferation, pyridoxal phosphate biosynthetic process, pyridoxine biosynthetic process, vitamin B6 metabolic process
PF4	platelet factor 4 (chemokine (C-X-C	chemokine activity, heparin	cytokine and chemokine mediated signaling pathway, immune response,

	motif) ligand 4)	binding	leukocyte chemotaxis, negative regulation of angiogenesis, negative regulation of megakaryocyte differentiation, platelet activation
PIK3CB	phosphoinositide-3-kinase, catalytic, beta polypeptide	1-phosphatidylinositol-3-kinase activity, binding, inositol or phosphatidylinositol kinase activity, insulin receptor substrate binding, phosphatidylinositol-4,5-bisphosphate 3-kinase activity, phosphotransferase activity, alcohol group as acceptor, transferase activity	G-protein coupled receptor protein signaling pathway, activation of MAPK activity, cellular calcium ion homeostasis, chemotaxis, embryonic cleavage, homophilic cell adhesion, phosphoinositide phosphorylation, phosphoinositide-mediated signaling, platelet activation, regulation of cell-matrix adhesion, signal transduction
PIP5K1B	phosphatidylinositol-4-phosphate 5-kinase, type I, beta	1-phosphatidylinositol-4-phosphate 5-kinase activity, kinase activity, protein binding, transferase activity	phosphatidylinositol metabolic process, phosphorylation
PKLR	pyruvate kinase, liver and RBC	magnesium ion binding, potassium ion binding, pyruvate kinase activity, pyruvate kinase activity, transferase activity	glycolysis, glycolysis, response to other organism
PKP2	plakophilin 2	binding, protein binding, structural molecule activity	cell-cell adhesion
PLA2G4A	phospholipase A2, group IVA (cytosolic, calcium-dependent)	calcium ion binding, hydrolase activity, lysophospholipase activity, phospholipase A2 activity	icosanoid metabolic process, lipid catabolic process, metabolic process, phospholipid catabolic process, platelet activating factor biosynthetic process
PLCG2	phospholipase C, gamma 2 (phosphatidylinositol-specific)	calcium ion binding, hydrolase activity, phosphoinositide phospholipase C activity, protein binding, signal transducer activity	intracellular signaling cascade, lipid catabolic process
PLD1	phospholipase D1, phosphatidylcholine-specific	hydrolase activity, phosphoinositide binding, phospholipase D activity, protein binding	Ras protein signal transduction, cell communication, chemotaxis, lipid catabolic process, metabolic process
PPBP	pro-platelet basic protein (chemokine (C-X-C motif) ligand 7)	chemokine activity, glucose transmembrane transporter activity, growth factor activity	cell proliferation, chemotaxis, defense response to bacterium, glucose transport, immune response
PRRG4	proline rich Gla (G-carboxyglutamic acid) 4 (transmembrane)	calcium ion binding, molecular_function	biological_process

PSTPIP1	proline-serine-threonine phosphatase interacting protein 1	catalytic activity, protein binding	cell adhesion, signal transduction
PTAFR	platelet-activating factor receptor	phospholipid binding, platelet activating factor receptor activity, receptor activity	G-protein coupled receptor protein signaling pathway, chemotaxis, cytokine production, immune response, inflammatory response, phosphoinositide-mediated signaling, signal transduction
PTX3	pentraxin-related gene, rapidly induced by IL-1 beta	zymosan binding	inflammatory response, opsonization, positive regulation of nitric oxide biosynthetic process, positive regulation of phagocytosis, response to yeast
PYGL	phosphorylase, glycogen; liver (Hers disease, glycogen storage disease type VI)	AMP binding, ATP binding, bile acid binding, drug binding, glucose binding, glycogen phosphorylase activity, nucleotide binding, phosphorylase activity, protein homodimerization activity, purine binding, pyridoxal phosphate binding, transferase activity, transferring glycosyl groups	carbohydrate metabolic process, glucose homeostasis, glycogen metabolic process
QPCT	glutaminyl-peptide cyclotransferase (glutaminyl cyclase)	acyltransferase activity, glutaminyl-peptide cyclotransferase activity, metal ion binding, peptidase activity, transferase activity, zinc ion binding	protein modification process, proteolysis
RAB27A	RAB27A, member RAS oncogene family	GTP binding, GTPase activity, nucleotide binding	protein transport, small GTPase mediated signal transduction
RBP7	retinol binding protein 7, cellular	lipid binding, retinal binding, retinol binding, transporter activity	transport
REPS2	RALBP1 associated Eps domain containing 2	calcium ion binding, calcium ion binding, protein binding	epidermal growth factor receptor signaling pathway, protein complex assembly
RHAG	Rh-associated glycoprotein	ammonium transmembrane transporter activity, ammonium transmembrane transporter activity, ankyrin binding, molecular_function, phosphopantetheine binding	ammonium transport, ammonium transport, blood circulation, cellular ion homeostasis, transport

RHD	Rh blood group, D antigen	molecular_function	biological_process
RNF217	ring finger protein 217	ligase activity, metal ion binding, protein binding, ubiquitin-protein ligase activity, zinc ion binding	protein ubiquitination during ubiquitin-dependent protein catabolic process, ubiquitin cycle
ROGDI	rogdi homolog (Drosophila)		
RXRA	retinoid X receptor, alpha	ligand-regulated transcription factor activity, metal ion binding, molecular_function, protein binding, retinoid-X receptor activity, sequence-specific DNA binding, steroid binding, steroid hormone receptor activity, tRNA-pseudouridine synthase activity, transcription coactivator activity, transcription factor activity, zinc ion binding	biological_process, positive regulation of transcription from RNA polymerase II promoter, regulation of transcription, DNA-dependent, signal transduction, transcription, vitamin metabolic process
SDPR	serum deprivation response (phosphatidylserine binding protein)	phosphatidylserine binding, protein binding	
SEC14L4	SEC14-like 4 (S. cerevisiae)	lipid binding, transporter activity	transport
SERPINB10	serpin peptidase inhibitor, clade B (ovalbumin), member 10	protein binding, serine-type endopeptidase inhibitor activity	
SGMS2	sphingomyelin synthase 2	ceramide cholinephosphotransferase activity, kinase activity, sphingomyelin synthase activity, transferase activity	lipid metabolic process, sphingolipid metabolic process, sphingomyelin biosynthetic process
SIGLEC5	sialic acid binding Ig-like lectin 5	protein binding, sugar binding	cell adhesion
SIRPA	signal-regulatory protein alpha		cell adhesion
SIRPB1	signal-regulatory protein beta 1		cell surface receptor linked signal transduction
SLC22A15	solute carrier family 22, member 15	transporter activity	transport
SLC22A4	solute carrier family 22 (organic cation/ergothioneine	ATP binding, DNA binding, carnitine transporter activity, ion transmembrane transporter	body fluid secretion, carnitine transport, ion transport, nucleosome assembly, organic cation transport,

	transporter), member 4	activity, nucleotide binding, secondary active organic cation transmembrane transporter activity, sodium ion binding, symporter activity, transporter activity	sodium ion transport
SLC2A5	solute carrier family 2 (facilitated glucose/fructose transporter), member 5	fructose transmembrane transporter activity, fructose transmembrane transporter activity, glucose transmembrane transporter activity, substrate-specific transmembrane transporter activity, sugar:hydrogen symporter activity, transporter activity	carbohydrate metabolic process, carbohydrate transport, fructose transport, glucose transport, transmembrane transport
SLC31A1	solute carrier family 31 (copper transporters), member 1	copper ion binding, copper ion transmembrane transporter activity, copper ion transmembrane transporter activity	copper ion transport, copper ion transport, ion transport
SLPI	secretory leukocyte peptidase inhibitor	serine-type endopeptidase inhibitor activity	
SNTB1	syntrophin, beta 1 (dystrophin-associated protein A1, 59kDa, basic component 1)	actin binding, calcium ion binding, calmodulin binding	muscle contraction
STEAP3	STEAP family member 3	FAD binding, binding, copper ion binding, electron carrier activity, iron ion binding, metal ion binding	apoptosis, cell cycle, ion transport, iron ion transport, oxidation reduction
STX11	syntaxin 11	SNAP receptor activity	intracellular protein transport, membrane fusion, vesicle-mediated transport
SYNE1	spectrin repeat containing, nuclear envelope 1	actin binding, actin binding, lamin binding, structural molecule activity	Golgi organization and biogenesis, muscle cell differentiation, nuclear organization and biogenesis
TACSTD2	tumor-associated calcium signal transducer 2	receptor activity	cell proliferation, cell surface receptor linked signal transduction, response to stimulus, visual perception
TFF3	trefoil factor 3 (intestinal)		defense response, digestion
TLR4	toll-like receptor 4	lipopolysaccharide binding, protein binding, protein binding, transmembrane receptor activity, transmembrane receptor activity	I-kappaB kinase/NF-kappaB cascade, T-helper 1 type immune response, detection of fungus, inflammatory response, innate immune response, macrophage activation, negative

			regulation of osteoclast differentiation, positive regulation of interleukin-12 biosynthetic process, positive regulation of interleukin-12 biosynthetic process, positive regulation of interleukin-8 biosynthetic process, positive regulation of tumor necrosis factor biosynthetic process, signal transduction, signal transduction
TM6SF1	transmembrane 6 superfamily member 1	molecular_function	biological_process
TMEM144	transmembrane protein 144		
TMEM50B	transmembrane protein 50B	molecular_function	biological_process
TMEM55A	transmembrane protein 55A	hydrolase activity	
TMEM56	transmembrane protein 56		
TNFSF13	tumor necrosis factor (ligand) superfamily, member 13	cytokine activity, tumor necrosis factor receptor binding	immune response, positive regulation of cell proliferation, positive regulation of isotype switching to IgA isotypes, signal transduction
TPP1	tripeptidyl peptidase I	protein binding, subtilase activity, tripeptidyl-peptidase I activity, tripeptidyl-peptidase I activity, tripeptidyl-peptidase I activity	bone resorption, lipid metabolic process, nervous system development, peptide catabolic process, protein catabolic process, proteolysis
TPRG1L	tumor protein p63 regulated 1-like		
TRIM10	tripartite motif-containing 10	metal ion binding, protein binding, zinc ion binding, zinc ion binding	hemopoiesis
TSPAN7	tetraspanin 7		protein amino acid N-linked glycosylation
TUBB1	tubulin, beta 1	GTP binding, GTPase activity, nucleotide binding, structural molecule activity	microtubule-based movement, protein polymerization
TXNDC3	thioredoxin domain containing 3 (spermatozoa)	ATP binding, nucleoside diphosphate kinase activity	CTP biosynthetic process, GTP biosynthetic process, UTP biosynthetic process, cell differentiation, cell redox homeostasis, multicellular organismal development, spermatogenesis

UBAC1	UBA domain containing 1	protein binding	ubiquitin cycle
UGCGL2	UDP-glucose ceramide glucosyltransferase-like 2	UDP-glucose:glycoprotein glucosyltransferase activity, UDP-glucose:glycoprotein glucosyltransferase activity, protein binding, transferase activity, transferring glycosyl groups	protein amino acid glycosylation
UNQ464	GLPG464		
VCAM1	vascular cell adhesion molecule 1	protein binding	cell-cell adhesion
VPS37C	vacuolar protein sorting 37 homolog C (S. cerevisiae)		protein transport
VSTM1	V-set and transmembrane domain containing 1		
WIPI1	WD repeat domain, phosphoinositide interacting 1	androgen receptor binding, estrogen receptor binding, phosphatidylinositol 3-phosphate binding, receptor binding	autophagy, vesicle targeting, trans-Golgi to endosome
